# The Identity of Information: How Deterministic Dependencies Constrain Information Synergy and Redundancy

**DOI:** 10.3390/e20030169

**Published:** 2018-03-05

**Authors:** Daniel Chicharro, Giuseppe Pica, Stefano Panzeri

**Affiliations:** 1 Department of Neurobiology, Harvard Medical School, Boston, MA 02115, USA; 2 Neural Computation Laboratory, Center for Neuroscience and Cognitive Systems@UniTn, Istituto Italiano di Tecnologia, Rovereto (TN) 38068, Italy

**Keywords:** information theory, mutual information decomposition, synergy, redundancy, 94A15, 94A17

## Abstract

Understanding how different information sources together transmit information is crucial in many domains. For example, understanding the neural code requires characterizing how different neurons contribute unique, redundant, or synergistic pieces of information about sensory or behavioral variables. Williams and Beer (2010) proposed a partial information decomposition (PID) that separates the mutual information that a set of sources contains about a set of targets into nonnegative terms interpretable as these pieces. Quantifying redundancy requires assigning an identity to different information pieces, to assess when information is common across sources. Harder et al. (2013) proposed an identity axiom that imposes necessary conditions to quantify qualitatively common information. However, Bertschinger et al. (2012) showed that, in a counterexample with deterministic target-source dependencies, the identity axiom is incompatible with ensuring PID nonnegativity. Here, we study systematically the consequences of information identity criteria that assign identity based on associations between target and source variables resulting from deterministic dependencies. We show how these criteria are related to the identity axiom and to previously proposed redundancy measures, and we characterize how they lead to negative PID terms. This constitutes a further step to more explicitly address the role of information identity in the quantification of redundancy. The implications for studying neural coding are discussed.

## 1. Introduction

The characterization of dependencies between the parts of a multivariate system helps to understand its function and its underlying mechanisms. Within the information-theoretic framework, this problem can be investigated by breaking down into parts the joint entropy of a set of variables [1,2,3] or the mutual information between sets of variables [4,5,6]. These approaches have many applications to study dependencies in complex systems such as gene networks (e.g., [7,8,9]), neural coding and communication (e.g., [10,11,12]), or interactive agents (e.g., [13,14,15]).

An important aspect of how information is distributed across a set of variables concerns whether different variables provide redundant, unique or synergistic information when combined with other variables. Intuitively, variables share redundant information if each variable carries individually the same information carried by other variables. Information carried by a certain variable is unique if it is not carried by any other variables or their combination, and a group of variables carries synergistic information if some information arises only when they are combined. The presence of these different types of information has implications for example to determine how the information can be decoded [16], how robust it is to disruptions of the system [17], or how the variables’ set can be compressed without information loss [18].

Characterizing the distribution of redundant, unique, and synergistic information is especially relevant in systems neuroscience, to understand how information is distributed in neural population responses. This requires identifying the features of neural responses that represent sensory stimuli and behavioral actions [19,20] and how this information is transmitted and transformed across brain areas [21,22]. The breakdown of information into these different types of components can determine the contribution of different classes of neurons and of different spatiotemporal components of population activity [23,24]. Moreover, the identification of synergistic or redundant components of information transfer may help to map dynamic functional connectivity and the integration of information across neurons or networks [25,26,27,28].

Although the notions of redundant, unique, and synergistic information seem at first intuitive, their rigorous quantification within the information-theoretic framework has proven to be elusive. Synergy and redundancy have traditionally been quantified with the measure called interaction information [29] or co-information [30], but this measure does not quantify them separately, and the presence of one or the other is associated with positive or negative values, respectively. Synergy has also been quantified using maximum entropy models as the information that can only be retrieved from the joint distribution of the variables [1,31,32].

However, a recent seminal work of [33] introduced a framework, called Partial Information Decomposition (PID), to more precisely and simultaneously quantify the redundant, unique, and synergistic information that a set of variables (or primary sources) *S* has about a target *X*. This decomposition has two cornerstones. The first is the definition of a general measure of redundancy following a set of axioms that impose desirable properties, in agreement with the corresponding abstract notion of redundancy [34]. The second is the construction of a redundancy lattice, structured according to these axioms, which reflects a partial ordering of redundancies for different sets of variables [33].

The PID framework has been further developed by others (e.g., [35,36,37,38,39,40]). However, the properties that the PID should have continue to be debated [38,41]. In particular, properly quantifying redundancy is inherently difficult because it requires assigning an identity to different pieces of information. This is needed to assess when different sources carry the same information about the target. The work in [35] argued that the original redundancy measure of [33] quantifies only quantitatively equal amounts of information and not information that is qualitatively the same. They introduced a new axiom, namely the identity axiom, which states that, for the concrete case of a target that is a copy of two sources, redundancy should correspond to the mutual information between the sources, and thus vanish for independent sources. Several redundancy measures that fulfill the identity axiom have been subsequently proposed [35,36,37]. However, although this axiom imposes a necessary condition to capture qualitatively common information, the question of how to generally determine the identity of different pieces of information to assess redundancy has not yet been solved, and information identity criteria are implicit in the axioms and measures used. Furthermore, the identity axiom is incompatible with ensuring the nonnegativity of the PID terms when there are more than two sources (multivariate case). This was proven by [42] with a counterexample that involves deterministic target-source dependencies, just like the target-source copy example used to motivate the axiom.

In this work, we examine in more detail how assumptions on the assignment of information identity determine the properties of the PIDs. We study in a general way the form of the PID terms for systems with deterministic target-source dependencies. These dependencies are particularly relevant to address the question of information identity because they allow exploring the consequences of alternative assumptions about how target-source identity associations constrain the existence of information synergistic contributions. These target-source identity associations naturally occur for example when the same variable appears both as a source and in the target: if some piece of information is assumed to be only associated with a variable that appears both as a source and as part of the target, this identity association would imply that there is no need to combine that source with any other to retrieve that piece of information. In other words, the corresponding synergy should be zero. Importantly, the deterministic relationships between the target and sources allow us to analyze how information identity criteria constrain the properties of the PIDs without the need to rely on any specific definition of PID measures.

To formalize the effect of deterministic target-source dependencies on the PID terms, we enunciate and compare the implications of two axioms that propose two alternative ways in which deterministic dependencies can constrain synergistic contributions because of assumptions on target-source identity associations. These axioms impose constraints to synergy for any (possibly multivariate) system with deterministic target-source dependencies, while the identity axiom only concerns a particular class of bivariate systems. We prove that the fulfillment of these axioms implies the fulfillment of the identity axiom and that several measures that fulfill the identity axiom also comply with one of the synergy axioms in general [36], or at least for a wider class of systems [35,43] than the one addressed by the identity axiom. The proof of the existence of negative terms when adopting the identity axiom was based on a concrete counterexample [41,42,44]. Oppositely, the stricter conditions of our synergy axioms allow us to explain in general how negative PID terms result from the specific information identity criteria underlying these axioms. More concretely, we derive, specifically for each of the two axioms, general expressions for deterministic components of the PID terms, which occur in the presence of deterministic target-source dependencies.

The comparison of the two axioms allows us to better understand the role of information identity in the quantification of redundancy. When the target contains a copy of some primary sources, an important difference between the redundancy measures derived from the two axioms regards their invariance, or lack thereof, to a transformation that reduces the target by removing all variables within it that are deterministically determined by this copy. This transformation does not alter the entropy of the target, and thus, a redundancy measure not invariant under it depends on semantic aspects regarding the identity of the variables within the target. We discuss why, in contrast to the mutual information itself, the PID terms may not be invariant to this transformation and depend on semantic aspects, as a consequence of the assignment of identity to the pieces of information, which is intrinsic to the notion of redundancy. In particular, we indicate how the overall composition of the target can affect the identity of the pieces of information and also can determine the existence of redundancy for independent sources (mechanistic redundancy [35]). Furthermore, based on this analysis, we identify the minimal set of assumptions that when added to the original PID axioms [33,34] can lead to negative PID terms. We indicate that this set comprises the assumption of the target invariance mentioned above. Overall, we conclude that if the redundancy lattice of [33] is to remain as the backbone of a nonnegative decomposition of the mutual information, a new criterion of information identity should be established that is compatible with the identity axiom, considers the semantic aspects of redundancy and results in less restrictive constraints on synergy in the presence of deterministic target-source dependencies than the two synergy axioms herein studied. Alternatively, the redundancy lattice should have to be modified to preserve nonnegativity.

We start this work by reviewing the PIDs (Section 2). We then introduce two alternative axioms that impose constraints on the value of synergistic terms in the presence of deterministic target-source dependencies, following an information identity criterion based on target-source identity associations (Section 3). Using these axioms, we derive general expressions that separate each PID term into a stochastic and a deterministic component for the bivariate (Section 4.1) and trivariate (Section 5.1) case. We show how these axioms constitute two alternative extensions of the identity axiom (Section 4.2) and examine if several previously-proposed redundancy measures conform to our axioms (Section 4.3). We reconsider the examples used by [42], characterizing their bivariate and trivariate decompositions and illustrating how in general negative PID terms can occur as a consequence of the information identity criteria underlying the synergy axioms (Section 4.4 and Section 5.2). The comparison between the two axioms allows us to discuss the implications of using an information identity criterion that, in the presence of deterministic target-source dependencies, identifies pieces of information in the target by assuming that their identity is related to specific sources. More generally, we discuss how our results constitute a further step to more explicitly address the role of information identity in the quantification of information (Section 4.5, Section 4.6 and Section 5.3).

## 2. A Review of the PID Framework

The seminal work of [33] introduced a new approach to decompose the mutual information into a set of nonnegative contributions. Let us consider first the bivariate case. Assume that we have a target *X* formed by one variable or by a set of variables and two variables (primary sources) 1 and 2 from which we want to characterize the information about *X*. The work in [33] argued that the mutual information of each variable about the target can be expressed as:

(1) I(X;1)=I(X;1.2)+I(X;1\2),

and similarly for I(X;2). The term I(X;1.2) refers to a redundancy component between variables 1 and 2, which can be obtained either by observing 1 or 2 separately. The terms I(X;1\2) and I(X;2\1) quantify a component that is unique to 1 and to 2, respectively, that is, the information that can be obtained from one of the variables alone, but that cannot be obtained from the other alone. Furthermore, the joint information of 12 can be expressed as:

(2) I(X;12)=I(X;1.2)+I(X;1\2)+I(X;2\1)+I(X;12\1,2),

where the term I(X;12\1,2) refers to the synergistic information of the two variables, that is information that can only be obtained when combining the two variables. Therefore, given the standard information-theoretic chain rule equalities [45]:

(3) $$\begin{array}{cc} I(X;12) & =I(X;1)+I(X;2|1)\\ & =I(X;2)+I(X;1|2), \end{array}$$

the conditional mutual information I(X;2|1), that is the average information that 2 provides about *X* once the value of 1 is known, is decomposed as:

(4) I(X;2|1)=I(X;2\1)+I(X;12\1,2),

and analogously for I(X;1|2). Conditioning removes the redundant component, but adds the synergistic component so that conditional information is the sum of the unique and synergistic terms.

In this decomposition, a redundancy and a synergy component can exist simultaneously. The work in [33] showed that the measure of co-information [30] that previously had been used to quantify synergy and redundancy, defined as:

(5) C(X;1;2)=I(i;j)−I(i;j|k)=I(i;j)+I(i;k)−I(i;j,k)

for any assignment of {X,1,2} to {i,j,k}, corresponds to the difference between the redundancy and the synergy terms of Equation (2):

(6) C(X;1;2)=I(X;1.2)−I(X;12\1,2).

More generally, [33] defined decompositions of the mutual information about a target *X* for any multivariate set of variables *S*. This general formulation relies on the definition of a general measure of redundancy and the construction of a redundancy lattice. In more detail, to decompose the information I(X;S), [33] defined a *source A* as a subset of the variables in *S* and a collection α as a set of sources. They then introduced a measure of redundancy to quantify for each collection the redundancy between the sources composing the collection, and constructed a redundancy lattice, which reflects the relation between the redundancies of all different collections. Here, we will generically refer to the redundancy of a collection α by I(X;α). Furthermore, following [46], we use a more concise notation than in [33]: for example, instead of writing {1}{23} for the collection composed by the source containing variable 1 and the source containing variables 2 and 3, we write 1.23, that is, we save the curly brackets that indicate for each source the set of variables and we use instead a dot to separate the sources. We will also refer to the single variables in *S* as *primary* sources when we want to specifically distinguish them from general sources that can contain several variables.

The work in [34] argued that a measure of redundancy should comply with the following axioms:

- Symmetry: I(X;α) is invariant to the order of the sources in the collection.
- Self-redundancy: The redundancy of a collection formed by a single source is equal to the mutual information of that source.
- Monotonicity: Adding sources to a collection can only decrease the redundancy of the resulting collection, and redundancy is kept constant when adding a superset of any of the existing sources.

The monotonicity property allows introducing a partial ordering between the collections, which is reflected in the redundancy lattice. Self-redundancy links the lattice to the joint mutual information I(X;S) because at its top there is the collection formed by a single source including all the variables in *S*. Furthermore, the number of collections to be included in the lattice is limited by the fact that adding a superset of any source does not change redundancy. For example, the redundancy between the source 12 and the source 2 is all the information I(X;2). The set of collections that can be included in the lattice is defined as:

(7) $$A\left(S\right)=\{\alpha \in P\left(S\right)-\left\{\emptyset \right\}:\forall \;A_{i},A_{j}\in \alpha ,A_{i}⊈A_{j}\},$$

where P(S)−{∅} is the set of all nonempty subsets of the set of nonempty sources that can be formed from *S*. This domain reflects the symmetry axiom in that it does not distinguish the order of the sources. For this set of collections, [33] defined a partial ordering relation to construct the lattice:

(8) ∀α,β∈A(S),(α⪯β⇔∀B∈β,∃A∈α,A⊆B),

that is, for two collections α and β, α⪯β if for each source in β there is a source in α that is a subset of that source. This partial ordering relation is reflexive, transitive, and antisymmetric. In fact, the consistency of the redundancy measures with the partial ordering of the collections, that is that I(X;α)≤I(X;β) if α⪯β represents a stronger condition than the monotonicity axiom. This is because the monotonicity axiom only considers the cases in which α is obtained from β adding more sources (e.g., α=1.2.3 and β=1.2), while the partial ordering comprises also the removal of variables from sources (e.g., α=1.2 and β=1.23, or α=1 and β=12.13).

**Figure 1:**
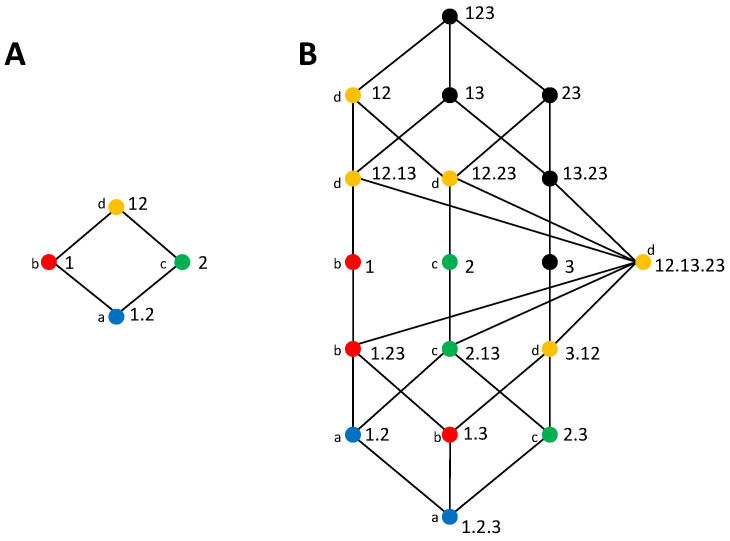
Redundancy lattices of [33]. The lattices reflect the partial ordering defined by Equation (8). (**A**) Bivariate lattice corresponding to the decomposition of I(X;12). (**B**) Trivariate lattice corresponding to the decomposition of I(X;123). The color and label of the nodes indicate the mapping of partial information decomposition (PID) terms from the trivariate to the bivariate lattice; in particular, nodes with the same color in the trivariate lattice are accumulated in the corresponding node in the bivariate lattice.

The mutual information multivariate decomposition was constructed in [33] by implicitly defining partial information measures Δ(X;α) associated with each node α of the redundancy lattice, such that redundancy measures are obtained from the sum of partial information measures:

(9) $$I\left(X;\alpha \right)=\sum_{\beta \in ↓\alpha }\Delta \left(X;\beta \right),$$

where ↓α refers to the set of collections lower than or equal to α in the partial ordering, and hence reachable descending from α in the lattice. The partial information measures are obtained inverting Equation (9) by applying the Möbius inversion to the terms in the lattice [33]. Redundancy lattices for *S* being bivariate and trivariate are shown in Figure 1. As studied in [46], a mapping exists between the terms of the trivariate and bivariate PIDs, as indicated by the colors and labels.

An extra axiom, called the identity axiom, was later introduced by [35] specifically for the bivariate redundancy measure:

- Identity axiom: For two sources $A_{1}$ and $A_{2}$, $I(A_{1}\cup A_{2};A_{1}.A_{2})$ is equal to $I(A_{1};A_{2})$.

The work in [35] pointed out that with the original measure of redundancy of [33] a nonzero redundancy is obtained for two independent variables and a target being a copy of them, while a measure quantifying the amount of qualitatively common information and not the quantitatively equal amount of information should be zero in this case. The work in [38] has specifically differentiated between the identity axiom, which states the form of redundancy for any degree of dependence between the primary sources when the target is a copy of them, and a more specific property, namely the independent identity property:

- Independent identity property: For two sources $A_{1}$ and $A_{2}$, $I\left(A_{1};A_{2}\right)=0\Rightarrow I(A_{1}\cup A_{2};$$A_{1}.A_{2})=\;0$.

This means that the independent identity property is fulfilled when the identity axiom is fulfilled, but the fulfilling of the independent identity property does not necessarily imply fulfilling the identity axiom. Several alternative measures have been proposed that fulfill the identity axiom [35,36,37]. The properties of the PID terms have been characterized, either based on the axioms and the structure of the redundancy lattice [46,47], or also considering the properties of specific measures [37,41,42,44,48,49]. However, only for specific cases such as multivariate Gaussian systems with univariate targets, it has been shown that several of the proposed measures are actually equivalent [50,51].

## 3. Stochasticity Axioms for Synergistic Information

In this section, we analyze the consequences of information identity criteria that in the presence of deterministic target-source dependencies identify pieces of information in the target by assuming that their identity is related to specific primary sources. As a first step, we formulate two axioms that impose constraints on synergistic information due to the presence of identity associations between variables within the target and the primary sources. Both axioms assume that, when a subset $X(S_{k})$ of the target *X* can be completely determined by one primary source $S_{k}$ from the set of primary sources $S=\{S_{1},\ldots ,S_{n}\}$, the identity of the bits of $X(S_{k})$ is associated with $S_{k}$. This target-source identity association between $X(S_{k})$ and $S_{k}$ then imposes constraints on the synergistic information that $S_{k}$ can provide about *X* combined with the other variables in *S*, because $S_{k}$ alone already can provide all the information about $X(S_{k})$. That is, the amount of synergy is constrained by the degree of stochasticity of the target variables with respect to the sources. The strength of the synergy constraints varies between the two axioms, as we will describe below, and we distinguish them as the weak and strong axiom. We start formulating the constraints that each axiom imposes on synergy conceptually, and subsequently, we will propose concrete constraints for the PID synergistic terms following from these axioms.

**Weak axiom on stochasticity requirements for the presence of synergy**: *Any primary source $S_{k}$ that completely determines a subset $X(S_{k})$ of variables of the target X does not provide information about $X(S_{k})$ synergistically, since $S_{k}$ alone provides all the information about $X(S_{k})$.*

**Strong axiom on stochasticity requirements for the presence of synergy:** *Any primary source $S_{k}$ that completely determines a subset $X(S_{k})$ of variables of the target X does not provide information about $X(S_{k})$ synergistically, since $S_{k}$ alone provides all the information about $X(S_{k})$. Furthermore, $S_{k}$ can only provide synergistic information about the rest of the target, $X-X(S_{k})$, to the extent that there is some remaining uncertainty in both $X-X(S_{k})$ and $S_{k}$ after determining $X(S_{k})$.*

Both axioms impose a common conceptual constraint on the presence of synergy, and the strong axiom imposes an extra constraint. The difference between the two axioms, as we will see in Section 4, can be understood in terms of the order in which $S_{k}$ is used to obtain information about the target. According to the weak axiom, there are no constraints on the synergistic contributions of $S_{k}$ to the information about $X-X(S_{k})$. Oppositely, the logic of the strong axiom is that, because $S_{k}$ already provides alone the information associated to the entropy $H(X\left(S_{k}\right))$, only if $H(X-X\left(S_{k}\right)|X\left(S_{k}\right))>0$ and $H(S_{k}|X\left(S_{k}\right)>0)$, then $S_{k}$ can still provide some extra information about *X*, and only in this case can this information possibly be synergistic.

The axioms constrain synergy on the basis that an identity is assigned to the bits of information related to the uncertainty $H(X\left(S_{k}\right))$ as corresponding to source $S_{k}$. In general, in the presence of dependencies between the variables constituting the target, bits cannot be associated univocally to specific variables within the target. Therefore, the identification of the bits of $H(X\left(S_{k}\right))$ with source $S_{k}$ does not follow univocally from the joint distribution of the variables. The assignment of an identity to the different pieces of information determines the assessment of whether different sources provide the same information and thus determines the quantification of redundant, unique, and synergistic information. This means that this quantification will also in general depend on the criterion used to assign identity, and will not be reducible to an analysis of the dependencies that are present in the joint distribution.

For simplicity, we will from now on refer to the axioms as the weak or strong stochasticity axioms, or simply the weak or strong axiom. In order to render these axioms operative, we have to formalize their conceptual formulation into sets of constraints imposed on the synergistic PID terms. We now propose the concrete formalization of the axioms. For the weak axiom, we will propose constraints on synergistic PID terms resulting from the existence of functional dependencies of target variables on primary sources (Section 3.1). For the strong axiom, we will also propose constraints resulting from these general functional dependencies, and moreover, we will propose extra constraints specific for the case in which some of the sources themselves are contained in the target (Section 3.2). Finally, we will briefly discuss the motivation to study these axioms in our subsequent analyses, namely as a way to examine how information identity criteria determine the PID terms (Section 3.3).

### 3.1. Constraints on Synergistic PID Terms That Formalize the Weak Axiom

We propose the following constraints to formalize the weak axiom:

**Constraints imposed by functional dependencies of target variables on primary sources:** *For a target X and a set of n variables (primary sources) $S=\{S_{1},\ldots ,S_{n}\}$, consider the subsets $X(S_{i})$ of X, i=1…n, such that $X(S_{i})$ can be determined completely by the single primary source $S_{i}$. Define $X^{\prime }={⋃}_{i}X\left(S_{i}\right)$ as the subset of X determined by single primary sources, then:*

(10) $$\Delta \left(X;\alpha \right)=\Delta \left(X-X^{\prime };\alpha \right)\;\;\forall \alpha \notin \underset{i\in S}{⋃}↓i,\;$$

*where ↓i indicates the collections reachable by descending the lattice from node i, corresponding to primary source $S_{i}$.*

The above means that the synergy about *X* is equal to the synergy in the lattice associated with the decomposition of the mutual information $I(X-X^{\prime };S)$ about a target $X-X^{\prime }$ that does not include the variables in $X^{\prime }$ determined by single primary sources alone. This implies that the primary sources cannot have synergistic information about a part $X^{\prime }$ of the target that is deterministically related to any of them. However, if we define $S^{\prime }$ as the subset of *S* comprising any primary source $S_{k}$ that determines some of the target variables (i.e., $S_{k}$ having a nonempty $X(S_{k})$), the weak axiom does not constrain that the variables in $S^{\prime }$ may provide information about other parts of the target in a synergistic way. Conversely, the strong axiom imposes that the variables in $S^{\prime }$ can only provide synergistic information to the extent that they are not themselves deterministically related to the variables in $X^{\prime }$.

### 3.2. Constraints on Synergistic PID Terms that Formalize the Strong Axiom

We propose the following constraints as a formalization of the strong axiom. First, we propose general constraints for any system with functional dependencies of target variables on primary sources:

**Constraints imposed by functional dependencies of target variables on primary sources:** *For a target X and a set of n variables (primary sources) $S=\{S_{1},\ldots ,S_{n}\}$, consider the subsets $X(S_{i})$ of X, i=1…n, such that $X(S_{i})$ can be determined completely by the single primary source $S_{i}$. Define $X^{\prime }={⋃}_{i}X\left(S_{i}\right)$ as the subset of X determined by single primary sources, then:*

(11) $$\Delta \left(X;\alpha \right)=\Delta \left(X-X^{\prime };\alpha |X^{\prime }\right)\;\;\forall \alpha \notin \underset{i\in S}{⋃}↓i.$$

That is, the synergy about *X* is equal to the synergy in the lattice associated with the decomposition of the mutual information $I(X-X^{\prime };S|X^{\prime })$ that *S* has about $X-X^{\prime }$ conditioned on $X^{\prime }$. Note that the PID of $I(X-X^{\prime };S|X^{\prime })$ is the same as the one of $I(X;S|X^{\prime })$, and thus, $\Delta \left(X-X^{\prime };\alpha |X^{\prime }\right)=\Delta \left(X;\alpha |X^{\prime }\right)$.

Comparing Equations (10) and (11), we can outline an important difference of the PIDs derived from each axiom. Define $X^{\prime \prime }$ as the variables in $X-X^{\prime }$ that can be determined as a function of $X^{\prime }$. Because in Equation (11) the synergistic PID terms are related to the decomposition of $I(X;S|X^{\prime })$, given the conditioning on $X^{\prime }$, these terms are invariant to a transformation of the target that removes from it all variables $X^{\prime \prime }$, i.e., $\Delta \left(X;\alpha \right)=\Delta \left(X-X^{\prime \prime };\alpha \right)$. Note that $I(X;S|X^{\prime })$ and also I(X;S) are themselves invariant to this transformation. Oppositely, according to Equation (10), the synergistic PID terms are related to the decomposition of $I(X-X^{\prime };S)$, which is not invariant to the removal of $X^{\prime \prime }$ from $X-X^{\prime }$. As we will discuss in detail in Section 4.6, the invariance, or lack thereof, to this transformation plays an important role in the characterization of the notion of redundancy that underpins the PIDs, and in particular determines the sensitivity of the PID terms to the overall composition of the target, comprising semantics aspects beyond the statistical properties of the joint distribution of the target variables.

In general, Equation (11) only expresses the synergistic terms of the PID of I(X;S) in terms of the synergistic terms of the PID of $I(X-X^{\prime };S|X^{\prime })$. However, these latter PID terms are themselves only specified after the definition of particular measures to implement the PID. However, in the more specific case where the primary sources in $S^{\prime }$ are in fact contained in the target (i.e., $X^{\prime }=S^{\prime }$), the logic of the conceptual formulation of the strong axiom leads to more specific constraints on the synergistic terms. In particular, because $X\left(S_{k}\right)=S_{k}$, then $H(S_{k}|X\left(S_{k}\right))=0$, and the primary sources in $S^{\prime }$ cannot provide other information about the target than the information about themselves. Since such information is already available without combining the sources in $S^{\prime }$ with any other source, this implies that the primary sources in $S^{\prime }$ do not provide any information about *X* synergistically. Therefore, we propose the following extra constraints for the synergistic PID terms specifically for the case in which $X^{\prime }=S^{\prime }$.

**Constraints imposed by copies of the primary sources within the target:** *For a target X and a set of n variables (primary sources) $S=\{S_{1},\ldots ,S_{n}\}$, consider the subset $X^{\prime }$ formed by all variables in X, which are a copy of one of the primary sources. Similarly, consider the subset $S^{\prime }$ formed by all primary sources with a copy within the target, i.e., $X^{\prime }=S^{\prime }$. Then:*

(12) $$\Delta \left(X;\alpha \right)=0\;\;\forall \alpha \notin \underset{i\in S}{⋃}↓i\;:\exists A\in \alpha ,S^{\prime }\cap A\neq \emptyset .$$

That is, there is no synergy for those nodes whose collection α has a source *A* containing a variable $S_{k}$ from $S^{\prime }$.

Since we have separately proposed the constraints of Equations (11) and (12) from the conceptual formulation of the strong axiom, they constitute, for the case of $X^{\prime }=S^{\prime }$, complementary requirements that should be fulfilled by a PID to be compatible with the strong axiom. However, we will show that for those previously proposed measures that at least for some class of systems comply with Equation (12), [35,36,43], Equation (11) is consistently fulfilled (Appendix D). More generally, the constraints of Equation (12) can be derived from Equation (11) in the case of $X^{\prime }=S^{\prime }$ if an extra desirable property is imposed to construct the PIDs (Appendix A). This extra property requires that the PID of $I(X;S|S^{\prime })$ is equivalent to the PID of $I(X;S-S^{\prime }|S^{\prime })$.

Furthermore, although the distinction between a *weak* and a *strong* axiom is motivated by the fact that the strong axiom conceptually imposes an extra requirement for the presence of synergy, this hierarchical relation is not conferred by construction to the concrete constraints imposed to the synergistic PID terms. The PIDs depend on the specific definition of the measures used to construct them, and these measures are expected to comply with one or the other axiom, so that PIDs complying with the axioms cannot be compared on the same measures. However, in agreement with the conceptual formulation, synergistic PID terms are expected to be smaller under the strong axiom because, in $\Delta (X-X^{\prime };\alpha |X^{\prime })$ (Equation (11)), for primary sources $S_{k}$ with a nonempty $X(S_{k})$, the synergy that other primary sources may have with $S_{k}$ will already be partially accounted by the combination of these other primary sources with $X(S_{k})$, which is part of $X^{\prime }$. See Appendix A for further details on the effect of conditioning on $X^{\prime }$ for the specific case of $X^{\prime }=S^{\prime }$.

### 3.3. Using the Stochasticity Axioms to Examine the Role of Information Identity Criteria in the Mutual Information Decomposition

In this work we will study how, based on the constraints imposed to the synergistic PID terms following the two stochasticity axioms, bivariate and trivariate PIDs are affected by deterministic relations between the target and the primary sources. Before focusing on that analysis, we complete the general formulation of the axioms with some considerations about their role in this work and their generality.

Regarding their role, we remark that we do not introduce the stochasticity axioms *per se*, to propose that they should be added to the set of axioms that PID measures should satisfy. Instead, the axioms are introduced to study the implications of identifying different pieces of information based on the target-source identity associations that result from deterministic target-source dependencies. The final objective is to better understand the role of information identity criteria in the quantification of redundancy. As we will see, these axioms are instrumental to characterize how the underlying information identity criterion can lead to negative PID terms. This characterization is also relevant in relation to previous studies because we prove that several previously proposed measures conform to the strong axiom generally [36] or at least for a wider class of systems [35,43] than the one concerned by the identity axiom.

Since our intention is not to formulate these axioms in their most general form, we have only considered their conceptual formulation, and propose concrete constraints to synergistic PID terms following from them, for the case in which there exist functional relations of target variables on single primary sources, that is, $X(S_{k})$. The same logic could be applied to formulate the axioms more generally and propose further constraints regarding functional relations of target variables on a subset of *S*. The PID terms affected by these other functional relations differ from the ones involved in Equations (10)–(12). For example, the existence of a functional relation X(1,2) depending on sources 1 and 2, would constrain the synergy of 12 with other variables, but not the synergy between 1 and 2. We will not pursue this more general formulation of synergy constraints. Conversely, to further simplify the derivations, we will focus on cases where the target *X* contains some of the primary sources themselves, that is, when the target overlaps with the sources as $X^{\prime }=S^{\prime }$. The more general formulation that considers target variables determined as a function of primary sources leads to the same main qualitative conclusions. All the general derivations in the rest of this work follow from the relations characteristic of the redundancy lattice (Equation (9)) and from the constraints to synergistic PID terms proposed following the axioms (Equations (10)–(12)). We do not need to select any specific measure of redundant, unique or synergistic information. For simplicity, from now on we will not distinguish between the conceptual formulation of the axioms and the constraints to synergistic PID terms proposed following from them, and we will refer to them as the weak and strong stochasticity axioms.

## 4. Bivariate Decompositions with Deterministic Target-Source Dependencies

We start with the bivariate case. Consider that the target *X* may have some overlap X∩12 with the sources 1 and 2. Following the weak stochasticity axiom (Equation (10)), synergy is expressed as:

(13) I(X;12\1,2)=I(X−12;12\1,2).

On the other hand, the strong stochasticity axiom (Equation (12)) implies that:

(14) $$I\left(X;12\backslash 1,2\right)=\left\{\begin{array}{c} I(X-12;12\backslash 1,2)\;\;\mathrm{if}\;X\cap 12=\emptyset \\ 0\;\;\mathrm{if}\;X\cap 12\neq \emptyset \end{array}\right..\;$$

From these expressions of the synergistic terms, we will now derive how deterministic relations affect the other PID terms.

### 4.1. General Formulation

For both forms of the stochasticity axiom, we will derive expressions of unique and redundant information in the presence of a target-source overlap. These derivations follow the same procedure: First, given that unique and synergistic information are related to conditional mutual information by Equation (4), the synergy stochasticity axioms determine the form of the unique information terms. Second, once the unique information terms are derived, their relation to the mutual information together with the redundancy term (Equation (1)) allows identifying redundancy. For both unique and redundant information terms this procedure separates the PID term into stochastic and deterministic components. These stochastic and deterministic components quantify contributions associated with the information that the sources provide about the non-overlapping part of the target, X−12, and the overlapping part, X∩12, respectively. However, how these components are combined depends on the order in which stochastic and deterministic target-source dependencies are partitioned. In particular, using the chain rule [45] of the mutual information, we can separate the information about the target in two different ways:

(15a) $$\begin{array}{cc} I(X;12) & =I(X-12;12)+I(X\cap 12;12|X-12) \end{array}$$

(15b) $$\begin{array}{cc} & =I(X\cap 12;12)+I(X-12;12|X\cap 12). \end{array}$$

The first case considers first the stochastic dependencies and after the conditional deterministic dependencies. In the second case, this order is reversed. We will see that for each axiom only one of these partitioning orders leads to expressions that additively separate stochastic and deterministic components for each PID term. Oppositely, the other partitioning order leads to cross-over components across PID terms, in particular to some PID terms being expressed in terms of the stochastic component of another PID term.

#### 4.1.1. PIDs with the Weak Axiom

We start with the PID of I(X;12) derived from the weak axiom (Equation (13)). Consider the mutual information partitioning order of Equation (15a), which can be re-expressed as:

(16) $$\begin{array}{c} I(X;12)=I(X-12;12)+H(X\cap 12|X-12), \end{array}$$

that is, the second summand corresponds to the conditional entropy of the overlapping target variables given the non-overlapping ones. We now proceed analogously for the PID terms. Since conditional mutual informations are the sum of a unique and a synergistic information component (Equation (4)), we have that:

(17) $$\begin{array}{cc} I(X;1\backslash 2) & =I(X;1|2)-I(X;12\backslash 1,2)\\ & =I(X-12;1|2)+I(X\cap 12;1|2,X-12)-I(X-12;12\backslash 1,2). \end{array}$$

The first equality indicates that unique information is conditional information minus synergy. The second equality uses the chain rule to separate the conditional mutual information stochastic and deterministic components, and applies the stochasticity axiom to remove the overlapping part of the target in the synergy term. Using again the relation between conditional mutual information and unique and synergistic terms (Equation (4)), but now, for the target X−12 we get:

(18) I(X;1\2)=I(X−12;1\2)+H(X∩1|2,X−12),

where we also used that I(X∩12;1|2,X−12) equals the entropy H(X∩1|2,X−12). Accordingly, the unique information of 1 can be separated into a stochastic component, the unique information about target X−12, and a deterministic component, the entropy H(X∩1|2,X−12). This last term is zero if the target does not contain source 1. If it does, it quantifies the entropy that only 1 as a source can explain about itself as part of the target, which is thus an extra contribution to the unique information.

Once we have identified the stochastic and deterministic components of the unique information we can use Equation (1) to characterize the redundancy. Combining Equations (1) and (18), we obtain:

(19) $$I\left(X;1.2\right)=I\left(X-12;1.2\right)+\left\{\begin{array}{c} 0\;\;\mathrm{if}\;X\cap 12=\emptyset \\ I(1;2|X-12)\;\;\mathrm{if}\;X\cap 12\neq \emptyset \end{array}\right..$$

Therefore, it suffices that one of the two primary sources overlaps with the target so that their conditional mutual information given the non-overlapping target variables contributes to redundancy. Note that when X∩12=∅ then X=X−12; hence, the axiom has no effect on the redundancy.

Following the same procedure, it is possible to derive expressions for the unique and redundant information terms, but applying the other mutual information partitioning order of Equation (15b). The resulting terms can be compared in Table 1 and are derived in more detail in Appendix B, where we also show the consistency between the expressions obtained with each partitioning order. We present in the upper part of the table the decompositions into stochastic and deterministic contributions for each PID term and for the two partitioning orders. To simplify the expressions, their form is shown only for the case of X∩i≠∅. With the alternative partitioning order, both the expressions of unique information and redundancy contain a cross-over component, namely the synergy about X−12, instead of being expressed in terms of the unique information and redundancy of X−12, respectively. Furthermore, the separation of the deterministic and stochastic components is not additive. This indicates that, while the chain rule holds for the mutual information, it is not guaranteed that the same type of separation holds separately for each PID term. Only for a certain partitioning order, when stochastic dependencies are considered first, unique and redundant information terms derived from the weak axiom can both be separated additively into a stochastic and a deterministic component without cross-over terms. We individuate in the lower part of the table the deterministic PID components obtained from the partitioning order for which each PID term is separated additively into a stochastic and deterministic component.

#### 4.1.2. PIDs with the Strong Axiom

The procedure to derive the unique and redundant PID terms is the same if the strong stochasticity axiom is assumed, but determining synergy with Equation (14) instead of Equation (13). To simplify the expressions we indicate in advance that if X∩12=∅ each PID term with target *X* is by definition equal to the one with target X−12 and we only provide expressions derived with some target-source overlap. In contrast to the weak axiom, with the strong axiom an additive separation of stochastic and deterministic components is obtained with the partitioning order of Equation (15b). See Appendix B for details about the other partitioning order. For the unique information the strong axiom implies that:

(20) $$I\left(X;1\backslash 2\right)=\left\{\begin{array}{c} I(X-12;1\backslash 2)+I(X-12;12\backslash 1,2)\;\;\mathrm{if}\;X\cap 1=\emptyset \\ H(1|2)\;\;\mathrm{if}\;X\cap 1\neq \emptyset \end{array}\right.,$$

and for the redundancy:

(21) I(X;1.2)=I(1;2).

As before, we summarize the PIDs in Table 2. Comparing Table 1 and Table 2, we see that the expressions obtained with the weak and strong axiom differ because of a cross-over contribution, corresponding to the synergy about X−12, which is transferred from redundancy to unique information. This is due to the synergy constraints imposed by each axiom: the strong axiom imposes that there is no synergy, and hence this part of the information has to be transferred to the unique information because the sum of synergy and unique information is constrained to equal the conditional mutual information. As a consequence, redundancy is reduced by an equivalent amount to comply with the constraints that relate unique informations and redundancy to mutual informations (Equation (1)). Furthermore, like for the weak axiom, the chain rule property does not generally hold for each PID term separately. This has been previously proven for specific measures. The work in [42] provided a counterexample for the original redundancy measure of [33] ($I_{min}$) and for the one of [35] ($I_{red}$). The work in [44] provided counterexamples for the redundancy and synergy measures of the decomposition based on maximum conditional entropy [36]. Our results prove this for any measure conforming to the stochasticity axioms. In particular, they show that the PID terms are consistent with the mutual information decompositions obtained applying the chain rule, but that, depending on the partitioning order and on the version of the axiom assumed, information contributions are redistributed between different PID terms, and between their stochastic and deterministic components.

### 4.2. The Relation between the Stochasticity Axioms and the Identity Axiom

In the previous section, we derived how the two stochasticity axioms imply different expressions for the redundancy term. We now examine how these expressions are related to the redundancy term stated by the identity axiom [35]. It is straightforward to show that the identity axiom is subsumed by both stochasticity axioms:

**Proposition** **1.** The fulfillment of the synergy weak or strong stochasticity axioms implies the fulfillment of the identity axiom.

**Proof.**  If X=12 then X∩12=12 and X−12=∅. If the weak stochasticity axiom holds, redundancy (Equation (19)) reduces to I(12;1.2)=I(1;2). If the strong stochasticity axiom holds, Equation (21) is already I(12;1.2)=I(1;2). ☐

Therefore, the stochasticity axioms represent two alternative extensions of the identity axiom: First, they do not only consider a target that is a copy of the primary sources, but a target with any degree of overlap or functional dependence with the primary sources. Second, they are not restricted to the bivariate case but are formulated for any number of primary sources. This means that their fulfillment imposes stricter conditions to the redundancy measures. Redundancy terms derived from each axiom coincide for the particular case that is addressed by the identity axiom, but more generally they differ. We will further discuss these differences below based on concrete examples.

### 4.3. How Different PID Measures Comply with the Stochasticity Axioms

We now investigate whether several proposed measures conform to the predictions of the stochasticity axioms. We examine the original redundancy measures of [33] ($I_{min}$), the one based on the pointwise common change in surprisal of [38] ($I_{ccs}$), the one based on maximum conditional entropy of [36] (SI), the one based on projected information of [35] ($I_{red}$), and the one based on dependency constraints of [43] ($I_{dep}$).

It is well-known that the redundancy measure $I_{min}$ does not comply with the identity axiom [35]. Even if I(1;2)=0, a redundancy $I_{min}\left(12;1.2\right)>0$ can be obtained. Nor does $I_{ccs}$ comply with the identity axiom. Since the fulfillment of the stochasticity axioms implies the fulfillment of the identity axiom, none of these measures complies with the stochasticity axioms.

On the other hand, SI, $I_{dep}$, and $I_{red}$ fulfill the identity axiom. We will show that SI always conforms to the strong stochasticity axiom. For $I_{dep}$, we will show that it complies with the strong axiom at least when some primary source is part of the target, i.e., X∩12≠∅. We will also show that $I_{red}$ complies with the strong axiom at least for the case of X∩12=12. This latter case is particularly relevant to examine nonnegativity (Section 5.3).

We proceed as follows: For SI, we now prove that it complies with Equation (12) specifically for the case in which some primary sources are also part of the target, which is the case considered throughout this work. The longer complete proof of compliance with Equation (11) for systems comprising any type of functional relation between parts of the target and single primary sources is left for Appendix C. For $I_{dep}$, we also provide here the proof of compliance with Equation (12) for the case of primary sources being part of the target. Again because of length reasons, the proof for $I_{red}$ is left for Appendix C. In all cases, we also prove in Appendix D that, for those cases herein studied in which these measures comply with Equation (12), Equation (11) is consistently fulfilled. We start with SI:

**Proposition** **2.** The PID associated with the redundancy measure SI [36] conforms to the synergy strong stochasticity axiom when some primary source is part of the target.

**Proof.**  Consider a target *X* and two sources 1 and 2. The redundancy measure SI is defined as:

(22) $$SI\left(X;1,2\right)=\max_{Q\in \Delta (p)}C_{Q}\left(X;1;2\right),$$

where the co-information $C_{Q}\left(X;1;2\right)$ is maximized within the family of distributions Δ(p) that preserves the marginals p(X,1) and p(X,2). We will now show that SI(X;1,2) conforms to Equation (21) when X∩12≠∅. It is a general property following from the definition of the co-information (Equation (5)) that, if either 1 or 2 are in *X*, that is, ∃i∈{1,2}:X∩i=i, then $C_{Q}\left(X;1;2\right)=I_{Q}\left(1;2\right)$. Further, because Δ(p) preserves p(X,1) and p(X,2), it suffices that X∩12≠∅ so that p(X,1,2) is preserved. This means that p(1,2) is preserved and $I_{Q}\left(1;2\right)=I\left(1;2\right)$ for all Q∈Δ(p). This leads to SI(X;1,2)=I(1;2). Given that for any valid bivariate PID one of the four PID terms already determines the other PID terms, because they have to comply with Equations (1) and (4), this shows that the PID equals the one derived from the strong axiom. ☐

We now continue with the proof for $I_{dep}$:

**Proposition** **3.** The PID associated with the redundancy measure $I_{dep}$ [43] conforms to the synergy strong stochasticity axiom when some primary source is part of the target.

**Proof.**  The work in [43] defined unique information based on the construction of a dependency constraints lattice in which constraints to maximum entropy distributions are hierarchically added. The unique information I(X;1\2) is defined as the least increase in the information $I_{Q}\left(X;12\right)$ when adding the constraint of preserving the distribution p(X,1) to the list of constraints imposed to the maximum entropy distribution *Q*. This results in the following expression for I(X;1\2), according to Appendix B of [43]:

(23) $$I\left(X;1\backslash 2\right)=\min\{I\left(X;1\right),I_{X1,X2}\left(X;1|2\right),I_{X1,X2,12}\left(X;1|2\right)\},$$

where $I_{X1,X2}\left(X;1|2\right)$ indicates the conditional mutual information for the maximum entropy distribution preserving p(X,1) and p(X,2), and analogously for $I_{X1,X2,12}\left(X;1|2\right)$. Now, consider that some source is part of the target, in particular, without loss of generality, that X∩1=1. In this case I(X,1)=H(1), and preserving p(X,2) and p(X,1) implies preserving the joint distribution p(X,1,2), given that 1 is part of *X*. This means that $I_{X1,X2}\left(X;1|2\right)=I_{X1,X2,12}\left(X;1|2\right)=I\left(X;1|2\right)$. Furthermore, I(X;1|2)=H(1|2). Since H(1|2)≤H(1) the unique information is I(X;1\2)=H(1|2), which already determines the PID, and in particular leads to the redundancy being I(X;1.2)=I(1;2). ☐

### 4.4. Illustrative Systems

**Figure 2:**
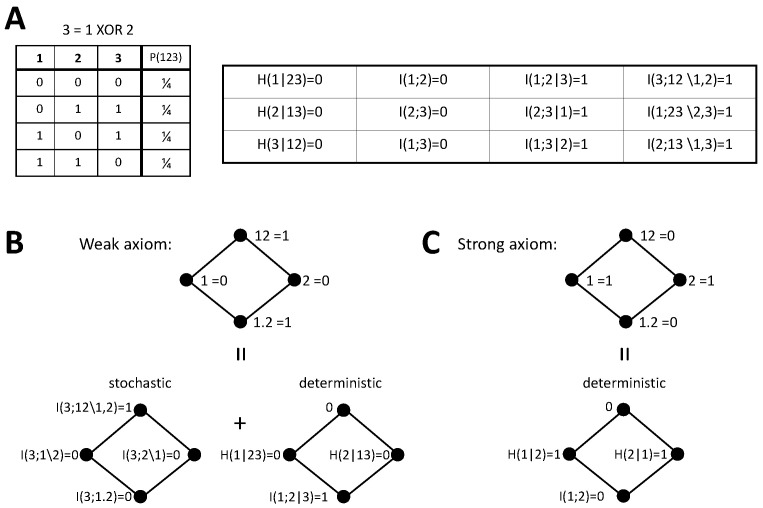
Bivariate decomposition of I(123;12) for the XOR system. (**A**) Joint distribution of the inputs 1 and 2 and the output 3 for the XOR operation. We also collect the value of the information-theoretic quantities used to calculate this bivariate decomposition and the trivariate decomposition I(123;123) in Section 5.2. (**B**) Bivariate decomposition derived from the weak stochasticity axiom. Stochastic and deterministic components are separated in agreement with Table 1. (**C**) Bivariate decomposition derived from the strong axiom. Only deterministic components are present, following Table 2.

**Figure 3:**
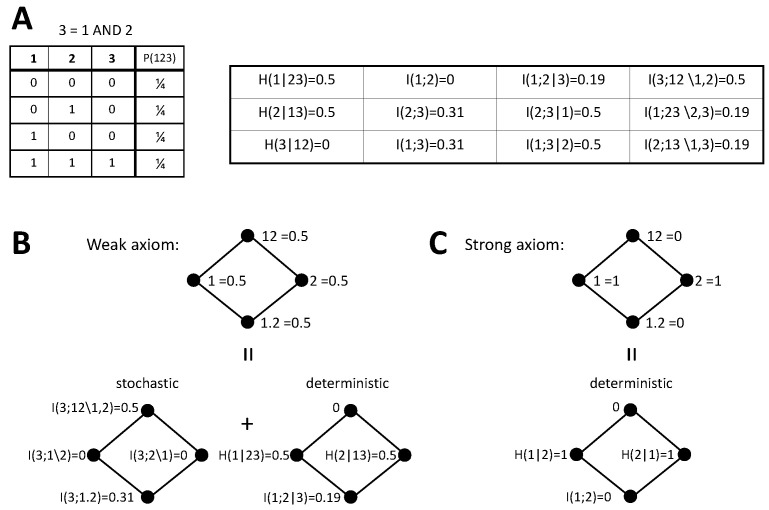
Bivariate decomposition of I(123;12) for the AND system. The structure of the figure is analogous to Figure 2. (**A**) Joint distribution of the inputs 1 and 2 and the output 3 for the AND operation. (**B**) Bivariate decomposition derived from the weak stochasticity axiom. (**C**) Bivariate decomposition derived from the strong axiom.

So far, we have derived the predictions for the PIDs according to each version of the stochasticity axiom, pointed out the relation with the identity axiom, and checked how different previously proposed measures conform to these predictions. We now analyze concrete examples to further examine the implications of our axioms on the PIDs. In particular, we reconsider two examples that have been previously studied in [42,44], namely the decompositions of the mutual information about a target jointly formed by the inputs and the output of a logical XOR operation or of an AND operation (see Figure 2A and Figure 3A, respectively). We first describe below the decompositions obtained, then in Section 4.5, we will discuss these decompositions in the light of the underlying assumptions on how to assign an identity to different pieces of information of the target. The deterministic components for these examples are derived without assuming any specific measure of redundancy, unique, or synergistic information. The stochastic components have already been previously studied and some of the terms depend on the measures selected to compute the PID terms. We will indicate previous work examining these terms when required.

#### 4.4.1. XOR

We first examine the XOR system. Consider an output variable 3 determined through the operation 3=1XOR2, resulting in the joint probability displayed in Figure 2A. We also indicate the values of the information-theoretic measures needed to calculate the PID bivariate decompositions studied here and that will also serve for the trivariate decompositions addressed in Section 5.2. We want to examine the decomposition of I(123;1,2), where the target is composed by the three variables. For each version of the stochasticity axiom we will focus on the mutual information partitioning order that allows separating additively a stochastic and a deterministic component of each PID term.

Since X−12=3, for the weak axiom the PID (Figure 2B) can be obtained by implementing the decomposition of I(3;12) and separately calculating the deterministic PID components $\Delta_{d}\left(123;\beta \right)$ as collected in Table 1. As indicated in [37], the decomposition of I(3;12) for the XOR operation can be derived without adopting any particular redundancy measure, just using Equations (1) and (4) and the axioms of [34] described in Section 2. Furthermore, the same value is obtained with $I_{ccs}$, which does not fulfill the nonnegativity axiom. There is no stochastic component of redundancy or unique information because I(3;i)=0 for i=1,2, and synergy contributes one bit of information. Regarding the deterministic components, redundancy has 1 bit because I(1;2|3)=1. The deterministic unique information components are zero because H(i|jk)=0 for i=1,2, and according to the axiom, there is no deterministic component of synergy.

In the case of the strong axiom (Figure 2C), since both primary sources overlap with the target, only deterministic components are larger than zero in the decomposition when selecting the partitioning order that additively separates stochastic and deterministic contributions, as indicated in Table 2. By assumption, there is no synergy. Since I(1;2)=0, the redundancy is also zero and all the information is contained in the unique information terms. As pointed out for the generic expressions, the two decompositions differ in the transfer of the stochastic component of synergy to unique information, which in turns forces an equivalent transfer from redundancy to unique information.

#### 4.4.2. AND

As a second example, we now consider the AND system. Following the weak axiom, again the decomposition can be obtained by implementing the PID of I(3;12) and separately calculating the deterministic PID components from Table 1, using the joint distribution of inputs and output displayed in Figure 3A. The PID of I(3;12) for the AND operation has also been already characterized and coincides for $I_{min}$ [33], $I_{red}$ [35], and SI [36]. However, in contrast to the XOR case, this decomposition depends on the redundancy measure used and for example differs for $I_{ccs}$. Each PID term contributes half a bit to I(123;12). Unique contributions come exclusively from the deterministic components. Each unique information amounts to half a bit because the output and one input determine the other input only when not both have a value of 0. Redundancy is also 0.5 bit, but it comes in part from a stochastic component and in part from a deterministic one. The stochastic component appears intrinsically because of the AND mechanism, even if the inputs are independent. This type of redundancy has been called mechanistic redundancy [35]. The deterministic component appears because, although the inputs are independent, conditioned on the output I(1;2|3)>0. The synergy I(3;12\1,2)=0.5 was also previously determined [33,35,36]. This PID differs from the one obtained with the weak axiom for the XOR example. Conversely, with the strong axiom the decomposition is the same as for the XOR example, because it is completely determined by I(1;2)=0. This latter decomposition is again in agreement with the arguments of [42,44] based on the identity axiom.

### 4.5. Implications of Target-Source Identity Associations for the Quantification of Redundant, Unique, and Synergistic Information

Each version of the stochasticity axiom implies a different quantification of redundancy. We now examine in more detail how these different quantifications are related to the notion of redundancy as common information about the target that can be obtained by observing either source alone. The key point is how identity is assigned to different pieces of information in order to assess which information about the target carried by the sources is qualitatively common to the sources. In particular, the logic of the strong axiom is that if a source is part of the target it cannot provide other information about the target than the information about itself. As a consequence, if the other source does not contain information about the former source, this information is unique. This logic rests on the assumption that when there is a copy of a primary source in the target we can identify and separate the bits of information about that copy from the information about the rest of the target. The idea of assigning an identity to bits of information in the target by associating them with specific variables also motivated the introduction of the identity axiom. Although this axiom was formulated for sources with any degree of dependence, its motivation [35] was mainly based on the case of independent sources, that is, the particular case considered by the independent identity property. For that case, we can identify the bits of information associated with variable 1 and the ones with variable 2, and thus redundancy, that should quantify the qualitatively equal information that is shared among the sources and not only common amounts of information, has to be null.

However, assigning an identity to pieces of information in the target is in general less straightforward. For example, in the XOR system, with target 123 and sources 1 and 2, we have two target-source identity associations, namely between each source and its copy in the target. However, the two bits of 123 cannot be identified as belonging to a certain variable, because of the conditional dependencies between the variables. The only information identity criterion that seems appropriate in this case to identify the two bits is the following: the bit that any first variable provides alone, and the bit that a second variable provides combined with the first. This lack of correspondence between pieces of information and individual variables is incompatible with the identification of the pieces of information based on the target-source identity associations that are formalized by the stochasticity axioms. To show this, we now consider different combinations of mutual information partitioning orders for I(123;1) and I(123;2) and show how, if the assignment of identity to the bits in the target 123 is based on target-source identity associations, the interpretation of redundant and unique information is ambiguous. First, consider that we decompose the information of each primary source as follows:

(24) $$\begin{array}{cc} I(123;1) & =I(1;1)+I(2;1|1)+I(3;1|12)=I(1;1)=H(1)\\ I(123;2) & =I(2;2)+I(1;2|2)+I(3;2|12)=I(2;2)=H(2). \end{array}$$

If we assume that we can identify the bit of information carried by each primary source about the target using the target-source identity associations, these decompositions would suggest that there is no redundant information. This is because each source only carries one bit of information about its associated copy within the target and I(1;2)=0 for the XOR system. However, keeping the same decomposition of I(123;1), we can consider alternative decompositions of I(123;2):

(25a) $$\begin{array}{cc} I(123;2) & =I(3;2)+I(1;2|3)+I(2;2|13)=I(1;2|3)=H(1) \end{array}$$

(25b) $$\begin{array}{cc} & =I(1;2)+I(3;2|1)+I(2;2|13)=I(3;2|1)=H(3). \end{array}$$

The redundancy and unique information terms should not depend on how we apply the chain rule to I(123;2). However, in contrast to Equation (24), the first decomposition of Equation (25a) suggests, based on the target-source identity associations, that there is redundancy between sources 1 and 2. In particular, I(123;2)=I(1;2|3) in Equation (25a) can be interpreted as information that source 2 provides about the copy of source 1 within the target, thus redundant with the information I(123;1) = I(1;1) in Equation (24) that source 1 has about its copy. The second decomposition in Equation (25b) further challenges the interpretation of redundancy and unique information based on the assignment of an identity to bits of information in the target given their association with the overlapping target variables. Given I(123;2)=I(3;2|1), source 2 provides information about 3. However, the bit of 3 is shared with the copies of 1 and 2 within the target, given the conditional dependencies of the XOR system. Moreover, the information in I(3;2|1) is information that source 2 provides about 3 after conditioning on the copy of source 1 within the target, so that the target-source identity association of 1 suggests that both sources are combined to retrieve this information. Note that for both I(1;2|3) and I(3;2|1) in Equation (25), we expressed the information in terms of the entropy of the target variable, 1 and 3, respectively, because it is the identity of the pieces of information within the target what determines their assignment to a certain PID term.

In summary, when using the target-source identity associations to identify pieces of information, different partitioning orders of the mutual information ambiguously suggest that the same information can be obtained uniquely, redundantly, or even in a synergistic way. These problems arise because, in contrast to the case of I(12;1,2) with independent sources, in the XOR system the two bits of 123 cannot be identified as belonging to a certain variable, and thus the target-source identity associations between the variables cannot identify the bits unambiguously.

The differences in the quantification of redundancy with each stochasticity axiom are related to the alternative interpretations of identity discussed for Equations (24) and (25). A notion of redundancy compatible with the weak axiom considers the common information about the target that can be obtained by observing either source alone or conditioned on variables in the target, which means that redundancy depends on the overall composition of the target. Indeed, the deterministic component of redundancy comprises the conditional dependence of the sources given the rest of the target, I(1;2|X−12), when there is a target-source overlap, and thus fits to Equation (25a), where the term I(1;2|3) appears. Conversely, with the strong axiom, when there is a target-source overlap, redundancy equals I(1;2) independently of X−12, in agreement with Equation (24). We will now further discuss the implications of this independence or dependence of redundancy on the overall composition of the target.

### 4.6. The Notion of Redundancy and the Identity of Target Variables

We showed above that enforcing the identification of the bits of 123 based on target-source identity associations between the variables leads to ambiguous interpretations of whether this information is retrieved redundantly, uniquely, or synergistically, depending on the partitioning order used to decompose the target. That is, the ambiguity arises because we consider 1, 2, and 3 as three separate variables within the target, which furthermore can be observed sequentially in any order, and not only simultaneously. The possibility to separately observe these variables is not relevant to quantify their entropy H(123) or the mutual information I(123;1,2), but, as we will argue below, it is potentially relevant to determine the PID terms.

In particular, for both the XOR and AND systems, 3 is completely determined by 12, so that H(123)=H(12) and I(123;1,2)=I(12;1,2). That is, the entropy and mutual information do not depend on whether we consider 3 as a separate variable or it is removed from the target. We can then ask how the assignment of the two bits to the PID terms depends on reducing the target 123 to 12. We repeat the comparison of different partitioning orders of I(123;1) and I(123;2) of Section 4.5 but now after this reduction. For I(12;1) the only possible partitioning orders are:

(26) $$\begin{array}{cc} I(12;1) & =I(1;1)+I(2;1|1)=I(1;1)=H(1)\\ & =I(2;1)+I(1;1|2)=I(1;1)=H(1), \end{array}$$

and analogously for I(12,2). Since I(1;2)=0, in all cases each source retrieves information about its associated copy in the target, and thus all information is contained in the unique information terms. Therefore, with the reduction of 123 to 12, the decompositions obtained are consistent with the ones derived from the strong axiom, which effectively also reduces 123 to 12 since the decomposition is independent of X−12 when 12 is part of the target. Indeed, [44] derived for the AND system the same decomposition as with the strong axiom using the measure SI and the reduction of 123 to 12.

The consistency between the strong axiom and the reduction of 123 to 12 is also reflected in the equality between the PIDs derived from the strong axiom for the XOR and AND systems. This is because the distributions of the targets 123 of these systems are isomorphic, i.e., one can be mapped to the other by relabeling the states, and are indistinguishable after the reduction to 12. However, the decompositions of the XOR and AND systems differ when derived with the weak axiom, and are not consistent with the reduction of 123 to 12. This is because with the weak axiom the PID terms have components in which 12 is explicitly separated from 3, in particular the redundancy contains the terms I(1;2|3) and I(3;1.2) (Table 1).

Therefore, an important difference between the redundancy measures derived from the two stochasticity axioms regards their invariance to transformations of the target consisting on the removal of the variables within it that are completely determined by copies of the primary sources contained in the target. We will in general call this type of invariance as TSC (target to sources copy) reduction invariance. Because the removal of these variables does not alter the entropy of the target, the mutual information is TSC reduction invariant. The lack of TSC reduction invariance implies that the redundancy depends on semantic aspects of the joint probability distribution of the target, related to the identity of the variables. The reason why the redundancy measure following from the weak axiom depends on these semantic aspects, while the measure following from the strong axiom does not, can be understood from how the identification of pieces of information based on the target-source variables associations is later used to constrain synergy in each case. With the weak axiom, the bits of $H(X\left(S_{k}\right))$ identified with the primary source $S_{k}$ due to the presence of $X(S_{k})$ within the target, are constrained to be non-synergistic in nature when the primary sources provide information about $X(S_{k})$. Oppositely, the weak axiom imposes no restriction on synergy about $X-X(S_{k})$. However, if there is some dependence between the variables $X(S_{k})$ and $X-X(S_{k})$ (i.e., $I(X\left(S_{k}\right);X-X\left(S_{k}\right))>0$), part of the bits of $X(S_{k})$ are shared by the variables $X-X(S_{k})$. This means that the same bits that are constrained to be non-synergistic in nature when the primary sources provide information about $X(S_{k})$ are still allowed to be synergistic when the primary sources provide information about $X-X(S_{k})$. Therefore, it is the identity of the variables about which the primary sources provide information what determines whether the same bits are subjected to the synergy constraints or not. The dependence of synergistic terms on the semantic aspects of the probability distribution determines that also redundancy terms inherit this dependence, because of their relation as terms of the PID. Oppositely, in the case of the strong axiom, once the identity of the bits of $H(X\left(S_{k}\right))$ is associated with the primary source $S_{k}$ due to the presence of $X(S_{k})$ within the target, the fact that due to $I(X\left(S_{k}\right);X-X\left(S_{k}\right))>0$ these bits can also be associated with $X-X(S_{k})$ is not considered, and they are constrained to be non-synergistic in nature without any consideration of the identity of the target variables about which the primary sources provide information.

The fact that redundancy is invariant or not to the TSC reduction has implications to determine other properties of the PIDs. In particular, it plays a crucial role in the counterexamples provided by [42,44] to prove that nonnegativity and left monotonicity are not compatible with the independent identity property. We will address in detail the counterexample of nonnegativity after studying trivariate PIDs with the stochasticity axioms, in Section 5. With regard to left monotonicity, it is useful to remind that [44] assumed the invariance of SI when reducing 123 to 12 to prove that left monotonicity is violated in the decomposition of I(123;1,2) of the AND system, because SI(3;1.2)>SI(123;1.2). As can be seen in Figure 3, although we have that I(3;1.2)>I(123;1.2) with the strong axiom, the opposite holds with the weak axiom, for which the invariance under reduction of 123 to 12 does not hold.

More generally, the TSC reduction is just one type of isomorphism of the target to which entropy and mutual information are always invariant. The comparison of the decompositions obtained with the two stochasticity axioms raises the question of whether we should expect the PIDs to be invariant to isomorphisms of the target, as the entropy and mutual information are. This question is intrinsically related to the role assigned to information identity in the notion of redundancy. Two aspects of this notion would justify a lack of invariance. First, the assessment of redundancy implies assigning an identity to pieces of information, and this identity can change depending on the variables included in the target. For example, for the target 123 in the XOR and AND systems, if 1, 2, and 3 are taken as variables that can be observed separately and sequentially, the bits of 123 cannot be identified as belonging to a certain variable, because of the conditional dependence I(1;2|3). However, after the reduction of 123 to 12, the two bits can be associated each to a single variable of the target because I(1;2)=0. Second, mechanistic redundancy can only be assessed when explicitly considering the mechanism of the input-output deterministic relation generating 3 from 12. This mechanism is not preserved under isomorphic transformations, and the information about it is lost when reducing 123 to 12 for the XOR and AND systems. These two arguments highlight the role of information identity to quantify redundancy, and indicate that requiring or not that the redundancy measures should be invariant to target isomorphisms implies further specifications of which is the underlying notion of redundancy that is quantified.

## 5. Trivariate Decompositions with Deterministic Target-Source Dependencies

We now extend the analysis to the trivariate case. This is particularly relevant because, in contrast to the bivariate case, it has been proven that, in the multivariate case, the PIDs that jointly comply with the monotonicity and the identity axioms do not guarantee the nonnegativity of the PID terms [42]. In particular, [42] used the XOR example we reconsidered above as a counterexample to show that PID terms can be negative. The work in [41] reexamined this counterexample indicating that the independent identity property, which is a weaker condition than the identity axiom, already implies the existence of negative terms. Therefore, we would like to be able to extend the general formulation of Section 4.1 to the trivariate case, and thus apply it to further examine the XOR and AND examples by identifying each component of the trivariate decomposition of I(123;123) and not only of the decomposition of I(123;12).

### 5.1. General Formulation

While in the bivariate lattice there is a single PID term that involves synergistic information, in the trivariate lattice of Figure 1B, all nodes that are not reached descending from 1, 2, or 3 imply by definition synergistic information, and the nodes of the form i.jk too. This is because these nodes correspond to collections containing sources composed by several primary sources, and hence quantify information only obtained by combining primary sources. The weak and strong axioms impose constraints on these terms given Equations (10) and (11), respectively.

#### 5.1.1. PIDs with the Weak Axiom

We begin with the weak stochasticity axiom, for a target *X* and three primary sources 1, 2, and 3. Expressing the general constraints of the weak axiom (Equation (10)) particularly for the trivariate case, and separating stochastic and deterministic components of the PID terms as in Table 1, that is, as $\Delta \left(X;\alpha \right)=\Delta \left(X-X^{\prime };\alpha \right)+\Delta_{d}\left(X;\alpha \right)$, Equation (10) can be expressed as:

(27) $$\Delta_{d}\left(X;\alpha \right)=0\;\;\forall \alpha \notin \underset{i=1,2,3}{⋃}↓i.$$

To characterize the remaining deterministic contributions to PID terms, analogously to the bivariate case, we apply the mutual information chain rule to separate stochastic and deterministic dependencies. Again we focus on the partitioning order that considers first the stochastic dependencies, since only this order leads to an additive separation of stochastic and deterministic components for each PID term. With this partitioning order, we obtain:

(28) $$\begin{array}{cc} I(X;123) & =I(X-123;123)+I(X\cap 123;123|X-123)\\ & =I(X-123;123)+H(X\cap 123|X-123). \end{array}$$

Following derivations analogous to the ones of Section 4.1 (see Appendix E), if a certain primary source *i* does not overlap with the target, the nodes that can only be reached descending from its corresponding node will not have a deterministic component. Accordingly, deterministic contributions are further restricted by:

(29) $$\Delta_{d}\left(X;\alpha \right)=0\;\;\forall \alpha \notin \underset{i\in X\cap \{1,2,3\}}{⋃}↓i.$$

This can be understood examining the term H(X∩123|X−123) in Equation (28). For example, suppose that the target includes 1 and 2 but not 3. Then the entropy in Equation (28) is H(12|X−123), corresponding to I(12;123|X−123). The PID terms that can be reached descending from 3 and not from 1 or 2 are Δ(X;3) and Δ(X;3.12) (see Figure 1B). The first quantifies information that can only be obtained from 3, and not from 12. The second is information that can be obtained from 3 or from 12, but not from 1 or 2 alone. However, the information I(12;123|X−123) can be obtained from either 1 or 2 alone, so there is no information exclusive of 12. This means that Δ(X;3) and Δ(X;3.12) do not contribute to the decomposition of H(12|X−123).

Using the condition of Equation (29), we can use the same procedure as in Section 4.1 to derive the expressions of all the deterministic PID trivariate components. These terms are collected in Table 3 and we leave the detailed derivations and discussion for Appendix E. Their expressions are indicated for the case in which variable *i* is part of the target and are symmetric with respect to *j* or *k* when this symmetry is characteristic of a certain PID term, or vanish otherwise, consistently with Equation (29).

The first two terms $\Delta_{d}\left(X;i\right)$ and $\Delta_{d}\left(X;i.jk\right)$ are nonnegative, the former because it is an entropy and the latter because according to the axiom adding a new source can only reduce synergy. However, for the terms $\Delta_{d}\left(X;i.j\right)$ and $\Delta_{d}\left(X;i.j.k\right)$ it is not guaranteed that they are nonnegative. For $\Delta_{d}\left(X;i.j\right)$, we will see examples of negative values below. For $\Delta_{d}\left(X;i.j.k\right)$, the conditional co-information can be negative if there is synergy between the primary sources when conditioning on the non-overlapping target variables, and this can happen when there is no synergy about the target, leading to a negative value. Therefore, following the weak stochasticity axiom, the PID cannot ensure the nonnegativity of all terms when deterministic target-source dependencies are in place. We will further discuss this limitation after examining the full trivariate decomposition for the XOR and AND examples.

#### 5.1.2. PIDs with the Strong Axiom

With the strong axiom, not only deterministic but stochastic components of synergy are restricted. There cannot be any synergistic contribution that involves a source overlapping with the target. Equation (12) can be applied with S=123. Furthermore, since synergistic terms have to vanish not only for the terms Δ(X;α) of the trivariate lattice but also of any bivariate lattice associated with it, given the mapping of PID terms between these lattices (Figure 1), this implies that in the trivariate lattice also the PID terms of the form i.jk are constrained. There is only one case in which synergistic contributions can be nonzero if there is any target-source overlap for the trivariate case, and this is when only one variable overlaps. Consider that only variable 1 is part of the target. Since there cannot be any synergy involving 1, all synergistic PID terms contained in I(X;1|2), I(X;1|3), or I(X;1|23) have to vanish, and also Δ(X;2.13) and Δ(X;3.12). It can be checked that this includes all synergistic terms except Δ(X;23) and Δ(X;1.23). The former quantifies synergy about other target variables and the latter synergy redundant with the information of 1 itself. With more than one primary source overlapping with the target all synergistic terms have to vanish for the trivariate case.

Like for the weak axiom, we now leave the derivations for Appendix E. The PID deterministic terms are collected in Table 4, again for simplicity showing their expressions for the case in which *i* overlaps with the target. The form of the expressions respects the symmetries of each term. For example, if *j* instead of *i* overlaps with the target then $\Delta_{d}\left(X;i.j\right)=I\left(i;j|k\right)-\Delta_{d}\left(X;j.ik\right)$. Note however that, because $\Delta_{d}\left(X;j.ik\right)=0$ when *i* overlaps, if both *i* and *j* overlap then $\Delta_{d}\left(X;i.j\right)=I\left(i;j|k\right)$. See Appendix E for further details.

In comparison to the deterministic components derived from the weak axiom there are two differences: First, the lack of conditioning on X−ijk is due to the reversed partitioning order selected. Like for the bivariate case, the deterministic PID components are independent of the non-overlapping target variables when adopting the strong stochasticity axiom. Second, assuming the strong axiom the terms $\Delta_{d}\left(X;i.jk\right)$ can only be nonzero if *j* and *k* are not contained in the target and when more than one source overlaps all terms of the form $\Delta_{d}\left(X;i.jk\right)$ vanish. In that case it is clear that $\Delta_{d}\left(X;i.j.k\right)$ can be negative, since the co-information can be negative. Therefore, also the PID derived from the strong axiom does not ensure nonnegativity. We will now show examples of negative terms for both PIDs.

### 5.2. Illustrative Systems

We now continue the analysis of the XOR and AND examples by decomposing I(123;123). Since now X−123=∅ the decompositions are completely deterministic and are obtained calculating the PID components described in Table 3 and Table 4. Accordingly, given that deterministic and joint PID terms are equal, we will use Δ(X;β) instead of $\Delta_{d}\left(X;\beta \right)$ to refer to them. As discussed in Section 4.4, the decompositions of the XOR system can be derived without assuming any particular redundancy measure. For the AND system, according to Table 3 and Table 4, only the terms $\Delta_{d}\left(X;i.jk\right)$ require selecting a particular measure. As before we assign to these terms the value that is equally obtained with $I_{min}$, $I_{red}$, and SI.

#### 5.2.1. XOR

**Figure 4:**
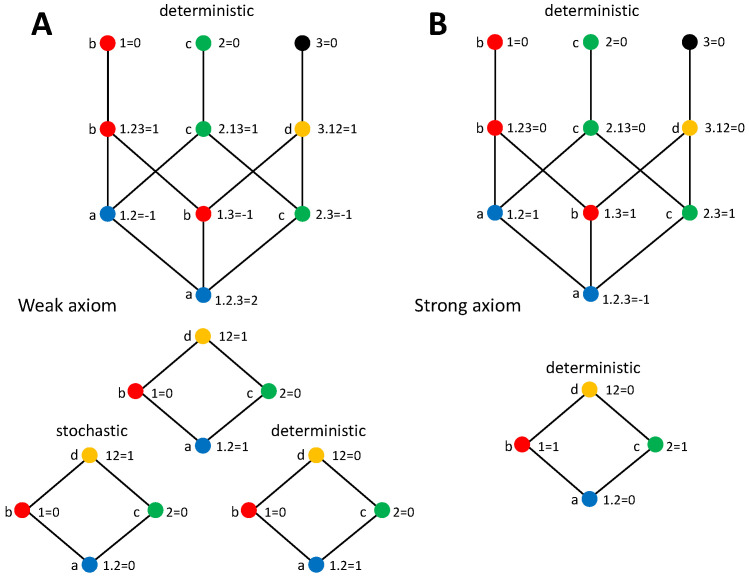
Trivariate decompositions of I(123;123) for the XOR system. (**A**) Decomposition derived from the weak stochasticity axiom. The trivariate redundancy lattice is displayed only for nodes lower than the single source nodes because all upper PID terms are zero. The bivariate decomposition of I(123;12) is shown again now indicating the mapping of the PID terms with colors and labels as in Figure 1. In particular, nodes with the same color in the trivariate lattice are accumulated in the corresponding node in the bivariate lattice. (**B**) Same as (**A**) but for the decomposition derived from the strong axiom.

We start with the XOR example and the decomposition derived from the weak stochasticity axiom (Figure 4A). We show the trivariate decomposition of I(123;123) and also again the decomposition of I(123;12), now indicating the mapping of the nodes with the trivariate decomposition. For the trivariate lattice we only show the nodes lower than the ones of the primary sources because for all others the corresponding terms are zero (Equation (27)). The PID terms are calculated considering Table 3 and the information-theoretic quantities displayed in Figure 2A.

The trivariate terms Δ(X;i) are all zero, because any two variables determine the third. This is also reflected in the terms Δ(X;i.jk) having 1 bit. The terms Δ(X;i.j) are all equal to −1 bit. These terms should quantify the redundant information between two variables, which is unique with respect to the third, but their interpretation is impaired by the negative values. Furthermore, Δ(X;i.j.k)=2, so that not only negative values exist but also the monotonicity axiom is violated, since I(X;i.j.k) > I(X;i.j). However, it can be verified that the values obtained are consistent from the point of view of the constraints linking PID terms and mutual informations (Equation (9)). Similarly, the calculated PID components are consistent between the bivariate and trivariate decompositions. In particular, the sum of the nodes with the same color or label in the trivariate lattice equals the corresponding node in the bivariate lattice. This equality holds for the joint bivariate lattice, and not for the deterministic lattice alone, even if in the trivariate case the lattice is uniquely deterministic. This reflects a transfer of stochastic synergy in the bivariate case to deterministic redundancy in the trivariate case (see yellow nodes labeled with *d*).

We now consider the decomposition derived from the strong axiom (Figure 4B). In this case also Δ(X;i) are all zero because any two variables determine the third, but now also Δ(X;i.jk) are zero. This is because the axiom assumes that there is no synergy involving any of the primary sources overlapping with the target. Δ(X;3.12)=0 is consistent with the lack synergy for the decomposition of I(123;12), as indicated by the mapping of the yellow nodes labeled with *d*. Furthermore, the mapping of all other PID terms is consistent. In particular, the 1 bit corresponding to the unique informations of the bivariate decomposition is contained in the terms Δ(X;i.j)=I(i;j|k) of the trivariate one. In comparison to the decomposition from the weak axiom, these terms are not negative, but instead, a negative value is obtained for Δ(X;i.j.k). Therefore nonnegativity is neither fulfilled for this decomposition.

We mentioned in Section 4.5 that, due to conditional dependencies, the only information identity criterion that seems appropriate for target 123 of the XOR system is to identify the two bits as follows: the bit that any first variable provides alone, and the bit that a second variable provides combined with the first. Oppositely, on one hand, the strong axiom assumes that each source alone can uniquely provide a bit, corresponding to its own identity, as reflected in the decomposition of I(123;12) (see Figure 4B). On the other hand, with the weak axiom, the second bit is classified as synergy, consistently with the idea that retrieving it requires the combination of two variables (Figure 4A). However, because the weak axiom still assumes that any information about an overlapping variable has to be redundant or unique, it imposes that the synergy is contained in the terms Δ(X;i.jk) in the trivariate decomposition and not in terms corresponding to nodes above the ones of single variables. Therefore, the weak axiom is still not compatible with that identification of the two bits as the one that can be obtained from a single variable and the one that can only be obtained from the combination of two variables.

#### 5.2.2. AND

**Figure 5:**
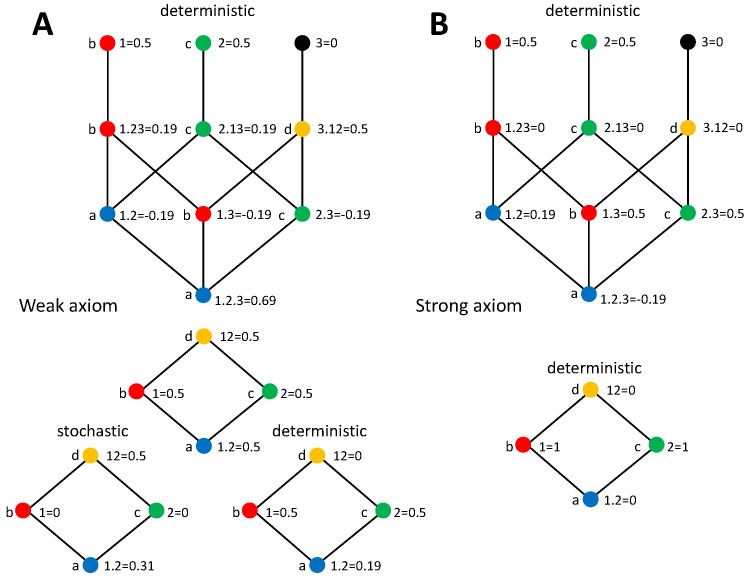
Trivariate decompositions of I(123;123) for the AND system. The structure of the figure is the same as in Figure 4. (**A**) Decompositions derived from the weak stochasticity axiom. (**B**) Decompositions derived from the strong axiom. Nodes with the same color in the trivariate lattice are accumulated in the corresponding node in the bivariate lattice.

We present the AND decomposition as a further example (Figure 5). All PID terms are derived using the information-theoretic quantities of Figure 3A in combination with Table 3 and Table 4. Like for the XOR case, the mapping of trivariate to bivariate decompositions is consistent. Again, both trivariate decompositions contain some negative term. With the strong axiom, while the bivariate decompositions for the XOR and AND example are equal because of the invariance reducing target 123 to 12, the trivariate PID terms differ substantially, reflecting the different symmetries of each operation. This is because in the trivariate decomposition 3 explicitly appears as a primary source and cannot be removed even if determined by 12.

### 5.3. PID Terms’ Nonnegativity and Information Identity

The decomposition of I(123;1,2,3) for the XOR system was used by [42,44] as a counterexample to show that with more than two sources there is no decomposition that can simultaneously comply with the monotonicity axiom and the identity axiom and also lead to global nonnegativity of the PID terms. The work in [41] recently pointed out that negative terms appear just when assuming the independent identity property, and not necessarily the identity axiom. However, the existence proofs in [41,42,44] only indicate that a negative term exists, without finding exactly which is the negative term and without determining all the PID terms (see Appendix F for more details).

Our results complement their proofs because the combination of the stochasticity axioms with the relations of the form of Equation (9) allows us to derive the complete PIDs, as shown in Table 3 and Table 4. The negative terms can be explained as a consequence of the deterministic components of the PID terms, which result from deterministic target-source dependencies. This is particularly relevant because, as proven in Section 4.3 and in the Appendix C, several proposed measures (i.e., SI, $I_{red}$, and $I_{dep}$) comply with the strong axiom (at least) when the primary sources are part of the target. Furthermore, the derivations from the stochasticity axioms relate the existence of negative terms to specific assumptions made to assign an identity to different pieces of information (Section 3 and Section 4.5). In more detail, the stochasticity axioms enforce that certain pieces of information are attributed to redundancy or unique information terms because of the target-source identity associations. As a consequence, deterministic components of the decomposition are bounded to the non-synergistic part of the redundancy lattice, which leads to negative terms in order to conform to the lattice structure and to the relations between PID terms and mutual informations (Equation (9)). Furthermore, as argued by [41], if the PID terms are to depend continuously on the probability distributions, the same problem of obtaining negative PID terms is expected to occur not only when deterministic target-source dependencies exist, but also in the limit of strong dependencies tending to be deterministic.

More generally, it is important to identify the minimal assumptions that, when added to the original core ingredients of [33,34], can lead to negative PID terms. These original ingredients are the three axioms of symmetry, self-redundancy, and monotonicity, and the relations of the measures in the redundancy lattice (Equation (9)). The work in [42,44] found that negative terms follow from adding the identity axiom, and [41] showed that they already follow from the weaker independent identity property. Furthermore, the comparison of the two stochasticity axioms and the discussion of information identity criteria (Section 4.6) allow a deeper appreciation of an extra assumption used in the proofs of [41,42,44] (see Appendix F), namely the TSC reduction invariance discussed in Section 4.6. This invariance was assumed, but not motivated in terms of what is expected from the notion of redundancy. Instead, it was assumed as inherited from the mutual information.

These two additional assumptions are less restrictive than adding the strong axiom, because the fulfillment of the strong axiom is a sufficient condition to fulfill the independent identity property (Section 4.2) and also to fulfill the TSC reduction invariance (Section 4.6). In contrast, the weak axiom does not imply this invariance. However, the decomposition of the XOR system derived from this axiom (Figure 4A) shows that it leads to negative terms, but also that it contradicts the monotonicity axiom. Therefore, if we want to preserve the original ingredients of the PID framework, the minimal additional assumptions that lead to negative terms are the independent identity property and the TSC reduction invariance.

We now assess these two assumptions in the light of the discussion of the role of information identity in the quantification of redundancy. Regarding the TSC reduction invariance, in Section 4.6, we indicated that this reduction can affect the identity of the pieces of information and remove information about the mechanisms that would result into mechanistic redundancy. This provides some arguments suggesting that the TSC reduction invariance should not be imposed to the redundancy measure. Regarding the independent identity property, when the target is a copy of two independent sources, the bits of information can be identified with each variable within the target and the target-source identity associations can be applied to assess that there is no redundancy. In fact, for the more general case of dependent sources, the identification of the bits is also consistent with the identity axiom. The bits are shared by the copies of the two sources in the target, and the target-source identity associations can be used to assess that redundancy equals the mutual information between the sources. Therefore, the considerations about information identity suggest that both the independent identity property and the identity axiom should be required. Altogether, this suggests that, from the two assumptions, only the independent identity property, and more generally the identity axiom, should be preserved.

We now review how several proposed redundancy measures comply or not with these two assumptions. SI, $I_{red}$, and $I_{dep}$ follow both assumptions (see [35,36,43], Section 4.3, and Appendix C). This leads to negative PID terms in the multivariate case. Oppositely, for the measures $I_{min}$ [33] and its simplification $I_{I}$ [42], it is straightforward to check that they fulfill the TSC reduction invariance, and nonnegativity has been proven [33], but they do not comply with the identity axiom [35]. This means that they only quantify common amounts of information, but not information that is qualitatively common to the sources. Furthermore, $I_{ccs}$ complies with the TSC reduction invariance, which it inherits from the co-information, but this measure was not defined to be nonnegative. Therefore, none of all these proposed redundancy measures complies with the independent identity property and does not comply with the TSC reduction invariance.

If the redundancy lattice and the axioms of [33,34] are to remain as the backbone of a nonnegative PID, we would require a new information identity criterion compatible with the identity axiom but leading to different assumptions about how deterministic target-source dependencies constrain the synergistic terms. The common assumption of the weak and strong axioms that information about an overlapping variable can only be redundant or unique may be too restrictive. As mentioned above, the analysis of how negative terms originate from the stochasticity axioms shows that they are produced by the accumulation of deterministic PID components in non-synergistic terms due to the constraints imposed based on target-source identity associations. Since the TSC reduction invariance only holds for the strong axiom, we expect that an information criterion that further relaxes these constraints can be compatible with the PID terms being non-TSC-invariants.

## 6. Discussion

### 6.1. Implications for the Theoretical Definition of Redundant, Synergistic and Unique Information

The proposal of [33] of decomposing mutual information into nonnegative redundant, unique, and synergistic components has been a fruitful and influential conceptual framework. However, a concrete implementation consistent with a set of axioms formalizing the notions for such types of information has proven to be elusive. The main difficulty stems from determining if redundant sources contain the same qualitative information, which requires assigning an identity to pieces of information in the target. The work in [35] pointed out that the redundancy defined by [33] only captures quantitatively the common amounts of information shared by the sources. They introduced the identity axiom to ensure that two independent variables cannot have redundant information about a copy of themselves. The lack of redundancy for this particular case has been enunciated as the independent identity property by [38]. However, Ref. [42] provided a counterexample showing that nonnegativity of the PID terms is not ensured when the identity axiom is assumed. This counterexample also involved a target constituted as a copy of the primary sources, in particular as the inputs and output variables of the XOR logical operation.

Although the identity axiom provides a necessary condition to capture qualitatively common information in the redundancy measure, previous studies have not directly examined how to assign an identity to different pieces of information in order to assess which information is redundant between sources. Since systems with deterministic target-source dependencies have been investigated for the introduction of the identity axiom [35] and to prove its incompatibility with the nonnegativity of the PID terms [36], here we systematically studied how specific information identity criteria constrain the PIDs of such systems. In particular, we examined the PIDs resulting from two information identity criteria that impose constraints on synergistic terms based on identity associations between the target and source variables. These associations result generically from deterministic target-source dependencies and, more concretely for the case we mainly studied, from the overlap between the target and the primary sources. We enunciated (Section 3) two stochasticity axioms that impose constraints of different strength on the synergistic terms. The weak axiom states that there cannot be synergistic information about the overlapping target variables. The strong axiom further constrains synergy assuming that the overlapping sources cannot provide other information than about themselves, and thus cannot contribute synergistic information about the non-overlapping part of the target.

We derived (Section 4.1) general formulas for the PID terms in the bivariate case, following each version of the stochasticity axiom. We showed that the PID terms can be separated into a stochastic and a deterministic component, which account for the information about the non-overlapping and overlapping target variables, respectively. We indicated that the stochasticity axioms subsume the identity axiom and provide two alternative extensions to characterize redundancy for any multivariate system with any degree of target-source overlap (Section 4.2). We showed that several previously proposed measures conform to the strong axiom either in general [36] or at least for classes of systems with target-source overlaps [35,43] wider than the ones considered by the identity axiom (Section 4.3). We then examined (Section 4.4) two concrete examples based on the XOR and AND logical operations, with variables 1 and 2 as inputs and variable 3 as output, calculating the PID of the mutual information I(123;12).

Using these examples, we showed how the identification of pieces of information based on target-source identity associations leads to an ambiguous determination of redundancy and unique information. This ambiguity is reflected in the possible selection of the two alternative stochasticity axioms, and is associated with different partitioning orders of the mutual information (Section 4.5). When using the weak axiom, each source can be combined with some target variables to provide information about other target variables, even in the presence of a target-source overlap. Conversely, the strong axiom assumes that any overlapping variable only provides information about itself, and thus redundant information is equal to the mutual information between the primary sources when there is some target-source overlap, independently of the non-overlapping target variables.

Therefore, a crucial difference between redundancy derived from the two axioms is its invariance to isomorphisms of the target. In the XOR and AND examples, since the output variable is completely determined by the inputs, the mutual information is invariant under the isomorphic reduction of the target 123 to 12, which we called the TSC (target to sources copy) reduction. This invariance of the mutual information holds without imposing any constraint on how the target variables are observed, either simultaneously or sequentially. However, it has been already shown that the PID terms can be sensitive to properties of the joint probability distribution of the target and sources to which the mutual information is not [52]. Here, we discussed two ways in which the overall composition of the target may affect redundancy (Section 4.6). First, the addition of new target variables can change the identity of the pieces of information associated with the previous variables by introducing new conditional dependencies. Second, the TSC reduction invariance is not compatible with the quantification of mechanistic redundancy [35], which has been recognized as the origin of redundancy between independent sources (e.g., [35,36,38,43]). This is because, in examples such as the XOR and AND systems with target 123, the TSC reduction erases the information about the mechanism generating the output variable 3, which is necessary to assess mechanistic redundancy. This means that the notion of redundancy itself depends on whether all the target variables can be observed separately or not. If it is accepted that redundancy should depend on the overall composition of the target, the corresponding redundancy measure cannot comply with the TSC reduction invariance and will depend on semantic aspects of the joint probability distribution of the target related to the identity of the variables.

In Section 5.1, we extended the general derivations to the trivariate case. This allowed us to understand what originates negative PID terms. While with the identity axiom the counterexample of [42] provides a proof of the existence of negative terms, under the stricter conditions of the stochasticity axioms, we could derive the complete PIDs, showing that several PID terms have a deterministic component that is not non-negatively defined (Section 5.2). This analysis is particularly relevant for the previously proposed measures that comply with the strong axiom for the XOR and AND systems with target 123 (Section 4.4). We have thus exposed the relation between the assumptions on information identity and the lack of nonnegativity. In particular, imposing that certain pieces of information can only be attributed to redundancy or unique information terms, based on the premise that their identity is associated with the sources, enforces that deterministic components of the mutual information are bounded to the non-synergistic part of the redundancy lattice. This leads to negative terms in order to conform to the lattice structure and to the lattice inherent relations between PID terms and mutual informations.

Although the notion of redundancy as information shared about the same pieces of information is intuitive in plain language, its precise implementation within the information-theoretic framework is not straightforward. The measure of mutual information has applications in many fields, such as communication theory and statistics [45]. Accordingly, a certain decomposition in terms of redundant, unique, and synergistic contributions may be compatible only with one of its interpretations. Indeed, if information is understood in the context of a communication channel [53], nonnegativity is required from its operational interpretation as the number of messages that can be transmitted without errors. Furthermore, semantic content cannot be attributed, and thus, information identity should rely only on the statistical properties of the distribution of the target variables. For example, in the case of the target composed by two independent variables, identity is assigned based on independence. Alternatively, if mutual information is used as a descriptor of statistical dependencies [54], nonnegativity is not required since locally negative information, or misinformation [55], simply reflects a certain change in the probability distribution of one variable due to conditioning on another variable. With this interpretation of information based on local dependencies, a criterion of information identity can introduce semantic content in association with the specific value of the variables, and common information of two sources can be associated with dependencies that induce coherent modifications of the probability distribution of the target variables [38]. These local measures of information may be interpreted operationally in terms of changes in beliefs, or in relation to a notion of information more associated with ideal observer analysis than with communication theory [55,56]. In this work, we have not considered local versions of mutual information, and we adopted the premise that nonnegativity is a desirable property for the PID terms.

With this aim, we identified the minimal extra assumptions that when added to the original axioms [33,34] lead to negative PID terms. Combining our analysis of the stochasticity axioms and the counterexample of [41,42,44], we pointed out that negativity appears due to the combined assumption of TSC reduction invariance and of the independent identity property (Section 5.3). Following our discussion of the role of information identity in the quantification of redundancy, we suggested that only the latter should be preserved in the search of a desirable redundancy measure.

### 6.2. Implications for Studying Neural Codes

Determining the proper criterion of information identity to evaluate when information carried by different sources is qualitatively common is essential to interpret the results of the PID in practical applications, such as in the analysis of the distribution of redundant, unique, and synergistic information in neural population responses. For example, when examining how information about a multidimensional sensory stimulus is represented across neurons, the decomposition should identify information about different features of the stimulus, and not only common amounts of information. The PID terms should reflect the functional properties of the neural population so that we can properly characterize the neural code. On the other hand, nonnegativity of the PID terms facilitates their interpretation not only as a description of statistical dependencies, but as a breakdown of the information content of neural responses, for example to assess the intersection information between sensory and behavioral choice representations [20,47,57].

The underlying criterion of information identity for the PID is also important when examining information flows among brain areas because, only if redundant and unique information terms correctly separate qualitatively the information, we can interpret the spatial and temporal dynamics of how unique new information is transmitted across areas. It is common to apply dynamic measures of predictability such as Granger causality [58] to characterize information flows between brain areas [21]. The effect of synergistic and redundant information components in the characterization of information flows with Granger causality has been studied [59,60], and ref. [61] applied their PID framework to decompose the information-theoretic measure of Granger causality, namely Transfer entropy [62,63], into terms separately accounting for state-independent and state-dependent components of information transfer. Furthermore, they also indicated which terms of the PIDs can be associated with information uniquely transmitted at a certain time or information transfer about a specific variable, such as a certain sensory stimulus [64]. These applications of the PID framework identify meaningful PID terms based on the redundancy lattice, and thus can be applied for any actual definition of the measures, but our considerations highlight the necessity to properly determine information identity in order to fully exploit their explanatory power.

Furthermore, our discussion of how the interpretation of information identity depends on the dependencies between the variables composing the target indicates that the analysis of how redundant, unique, and synergistic information components are distributed across neural population responses can be particularly useful in combination with interventional approaches [20,65]. In particular, the manipulation of neural activity with optogenetics techniques [66,67] can disentangle causal effects from other sources of dependencies such as common factors. Although this work illustrates the principled limitations of current PID measures, their combination with these powerful experimental techniques can help to better probe the functional meaning of the PID terms.

### 6.3. Concluding Remarks

We investigated the implications for the quantification of redundant, unique, and synergistic information of information identity criteria that, in the presence of deterministic target-source dependencies, assign an identity to pieces of information based on identity associations between the target and sources variables. Our analysis suggests that, if the redundancy lattice of [33] is to remain as the backbone of a nonnegative decomposition of the mutual information, a new criterion of information identity should be established that, while conforming to the identity axiom, it is less restrictive in the presence of deterministic target-source dependencies than the ones herein studied. 

## Figures and Tables

**Table 1:** Decompositions of synergistic, unique, and redundant information terms into stochastic and deterministic contributions obtained assuming the weak stochasticity axiom. For each term we show the decompositions resulting from two alternative mutual information partitioning orders (Equation (15)), which are consistent with each other (see Appendix B). For the partitioning order leading to an additive separation of each partial information decomposition (PID) term into a stochastic and deterministic component we also individuate the deterministic contributions $\Delta_{d}\left(X;\beta \right)$. Synergy has only a stochastic component, according to the axiom (Equation (13)). Expressions of unique information come from Equations (18) and (A3), and the ones of redundancy from Equations (19) and (A5). The expressions have been simplified with respect to the equations, indicating their form for the case X∩i≠∅. The terms $\Delta_{d}\left(X;\beta \right)$ have analogous expressions for X∩j≠∅ when a symmetry exists between *i* and *j* and are zero otherwise.

Term	Decomposition
I(X;ij\i,j)	I(X−ij;ij\i,j)
I(X;i\j)	$\begin{array}{c} I(X-ij;i\backslash j)+H(i|j,X-ij)\\ H(i|j)-I(X-ij;ij\backslash i,j) \end{array}$
I(X;i.j)	$\begin{array}{c} I(X-ij;i.j)+I(i;j|X-ij)\\ I(i;j)+I(X-ij;ij\backslash i,j) \end{array}$
**Term**	**Measure**
$\Delta_{d}\left(X;ij\right)$	0
$\Delta_{d}\left(X;i\right)$	H(i|j,X−ij)
$\Delta_{d}\left(X;i.j\right)$	I(i;j|X−ij)

**Table 2:** Decompositions of synergistic, unique, and redundant information terms into stochastic and deterministic contributions obtained assuming the strong stochasticity axiom. The table is analogous to Table 1. Synergy is null according to the axiom (Equation (14)). Expressions of unique information come from Equations (A8) and (20), and the ones of redundancy from Equations (A9) and (21). Again, expressions are shown for the case X∩i≠∅, with the corresponding symmetries holding for X∩j≠∅ and with terms $\Delta_{d}\left(X;\beta \right)$ equal to zero otherwise.

Term	Decomposition
I(X;ij\i,j)	0
I(X;i\j)	$\begin{array}{c} I(X-ij;i\backslash j)+I(X-ij;ij\backslash i,j)+H(i|j,X-ij)\\ H(i|j) \end{array}$
I(X;i.j)	$\begin{array}{c} I(i;j|X-ij)+I(X-ij;i.j)-I(X-ij;ij\backslash i,j)\\ I(i;j) \end{array}$
**Term**	**Measure**
$\Delta_{d}\left(X;ij\right)$	0
$\Delta_{d}\left(X;i\right)$	H(i|j)
$\Delta_{d}\left(X;i.j\right)$	I(i;j)

**Table 3:** Deterministic components of the PID terms for the trivariate decomposition derived from the weak stochasticity axiom. All terms not included in the table have no deterministic component due to the axiom. These expressions correspond to the case in which the primary source *i* overlaps with the target. If *i* does not overlap, $\Delta_{d}\left(X;i\right)$ and $\Delta_{d}\left(X;i.jk\right)$ are zero, while the other terms depend on their characteristic symmetry for the other variables *j* and *k*, and vanish if none of the variables with the corresponding symmetry overlaps with the target. See the main text and Appendix E for details.

Term	Measure
$\Delta_{d}\left(X;i\right)$	H(i|jk,X−ijk)
$\Delta_{d}\left(X;i.jk\right)$	I(X−jk;jk\j,k)−I(X−ijk;jk\j,k)
$\Delta_{d}\left(X;i.j\right)$	$I\left(i;j|k,X-ijk\right)-\left[\Delta_{d}\left(X;i.jk\right)+\Delta_{d}\left(X;j.ik\right)\right]$
$\Delta_{d}\left(X;i.j.k\right)$	$C\left(i;j;k|X-ijk\right)+\Delta_{d}\left(X;i.jk\right)+\Delta_{d}\left(X;j.ik\right)+\Delta_{d}\left(X;k.ij\right)$

**Table 4:** Deterministic components of the PID terms for the trivariate decomposition derived from the strong stochasticity axiom. All terms not included in the table have no deterministic component due to the axiom. Again, the expressions shown here correspond to the case in which the source *i* overlaps with the target. For $\Delta_{d}\left(X;i.jk\right)$ we further consider that neither *j* nor *k* overlap with the target, and otherwise this term vanishes. If *i* does not overlap, $\Delta_{d}\left(X;i\right)$ is zero, while the other terms depend on their characteristic symmetry for the other variables *j* and *k* and vanish otherwise. See the main text and Appendix E for details.

Term	Measure
$\Delta_{d}\left(X;i\right)$	H(i|jk)
$\Delta_{d}\left(X;i.jk\right)$	I(i;jk\j,k)
$\Delta_{d}\left(X;i.j\right)$	$I\left(i;j|k\right)-\Delta_{d}\left(X;i.jk\right)$
$\Delta_{d}\left(X;i.j.k\right)$	$C\left(i;j;k\right)+\Delta_{d}\left(X;i.jk\right)$

**Figure A1:**
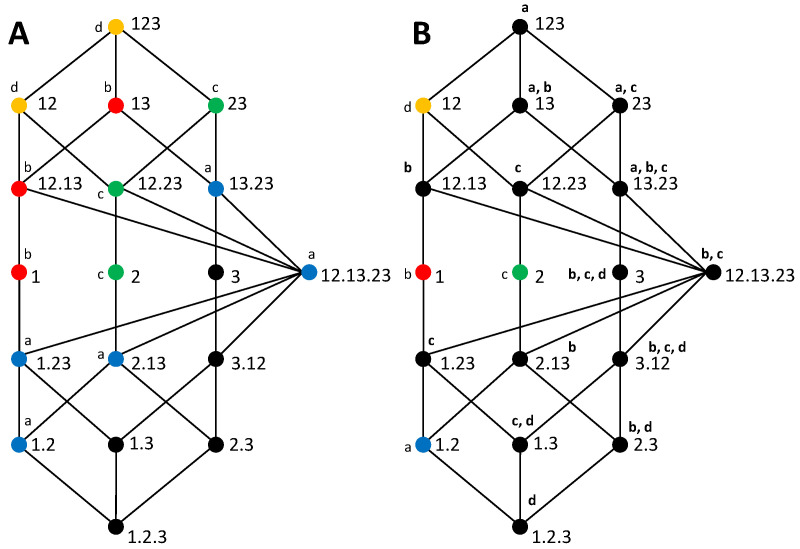
Redundancy lattices of the decomposition of I(X;123) (**A**) and of I(X;123|3) (**B**). (**A**) Black nodes correspond to PID terms decomposing I(X;3). Colored labeled nodes correspond to the decomposition of I(X;12|3). Nodes labeled with *a* contribute to the redundancy I(X;1.2|3), labeled with *b* to the unique information I(X;1\2|3), with *c* to the unique information I(X;2\1|3), and with *d* to synergy I(X;12\1,2|3). (**B**) Colored labeled nodes correspond to the decomposition of I(X;12|3). Colors and labels indicate the mapping to the same decomposition in the lattice of (**A**). Black nodes correspond to PID terms involved in the constraints of Equation (A1a–d). The bold letters refer to the subequations constraining each node.

## Appendix A. The Relations between the Constraints to Synergy Resulting from the Strong Axiom for the General Case of Functional Dependencies and for the Case of Sources Being Part of the Target

In Section 3, we argued that when any primary source $S_{k}$ that deterministically explains a subset $X(S_{k})$ of the target is in fact part of the target (i.e., $X^{\prime }=S^{\prime }$), the conceptual formulation of the strong axiom implies extra constraints that cancel certain synergistic terms (Equation (12)). These constraints specific of the case $X^{\prime }=S^{\prime }$ were not derived as a subcase of the general constraints associated with the strong axiom for any type of functional dependence (Equation (11)). Oppositely, we separately proposed these specific constraints following the logic of the strong axiom that indicates that any source that is part of the target cannot be involved in synergistic contributions because it can only contribute to provide the information about itself and it can do so without being combined with any other. We here examine the consistency of Equation (12) with Equation (11), showing that, if imposing an extra desirable property to the PID terms, Equation (12) can be derived from Equation (11). Without this further condition, the fulfillment of Equation (11) and of Equation (12) can be seen as separate requirements that should be fulfilled by a PID compatible with the strong axiom.

In general, Equation (11) only establishes a relation between the synergistic terms of the PID of I(X;S) and the synergistic terms of the PID of $I(X-X^{\prime };S|X^{\prime })$, or equivalently of $I(X;S|X^{\prime })$, as indicated in Section 3. When $X^{\prime }=S^{\prime }$, the synergistic terms of the decomposition of I(X;S) are expressed in terms of the synergistic terms of the decomposition of $I(X-S^{\prime };S|S^{\prime })$, or $I(X;S|S^{\prime })$. Both decompositions have to comply with the relations between mutual informations and partial informations of Equation (9). However, without any further assumption on how the PID terms should be defined, we cannot further specify the form of the PID terms $\Delta (X;S|S^{\prime })$. We will now show under which further conditions imposed to the PID terms $\Delta (X;S|S^{\prime })$ Equation (12) is obtained as a subcase of Equation (11).

Before addressing the general case, we start with an example to understand the constraints that Equation (9) imposes to the decomposition of $I(X;S|S^{\prime })$. In particular, consider three primary sources 1, 2, and 3 and a target $X=\{X-X^{\prime },3\}$, that is $X^{\prime }=S^{\prime }=3$. In this case, Equation (11) links the decomposition of I(X;123) to the decomposition of I(X;123|3). In Figure A1A,B, we show the redundancy lattices of I(X;123) and I(X;123|3), respectively. The PID terms of both lattices should be consistent with the equations that relate them to mutual information quantities (Equation (9)). In particular, using the chain rule to break down I(X;123|3), we have that:

(A1a) $$\begin{array}{cc} I(X;123|3) & =I(X;12|3)+I(X;3|12,3)=I(X;12|3)+0 \end{array}$$

(A1b) $$\begin{array}{cc} I(X;13|3) & =I(X;1|3)+I(X;3|1,3)=I(X;1|3)+0 \end{array}$$

(A1c) $$\begin{array}{cc} I(X;23|3) & =I(X;2|3)+I(X;3|2,3)=I(X;2|3)+0 \end{array}$$

(A1d) $$\begin{array}{cc} I(X;3|3) & =0. \end{array}$$

Note that the mutual information quantities in which 3 appears both as a source and as a target variable can only be related to the decomposition of I(X;123|3), while the others are related to both decompositions. In particular, given Equation (9), we should be able to recover, as sums of PID terms from any of the lattices, the measures I(X;12|3), I(X;1|3), and I(X;2|3). The other measures are only related to the PID of I(X;123|3). Because of the conditioning on the target variable 3, the source 3 cannot contribute any information, since H(3|3)=0. This is reflected in the fact that I(X;3|12,3), I(X;3|1,3), I(X;3|2,3), and I(X;3|3) are all zero. Given Equation (9), this imposes four constraints on the PID terms Δ(X;α|3), as sums of terms that should cancel.

Without any further assumption on the properties of these PID terms these constraints cannot determine the PID terms, since the cancellation could be achieved by a combination of positive and negative PID terms. However, if we assume the nonnegativity of the terms Δ(X;α|3), this implies that all PID terms summed by Equation (9) to obtain I(X;3|12,3), I(X;3|1,3), I(X;3|2,3), or I(X;3|3) should vanish. In Figure A1B we indicate the PID terms that vanish with black colored nodes and with bold letters referring to the set of sub-equations of Equation (A1) that imply their cancellation. For example, from Equation (9), the fact that I(X;3|1,3)=0 implies that PID terms of Figure A1B associated with the nodes in (↓13)−(↓1) have to vanish. These are the nodes that can be reached descending from 13 but that cannot be reached descending from 1. Similarly, I(X;3|12,3)=0 indicates that the terms associated with nodes in (↓123)−(↓12) have to vanish. Each of the four constraints cancels a set of PID terms identified as (↓{W,3})−(↓W), where $W\subseteq S-S^{\prime }$.

Altogether, given the constraints of Equation (A1), only the PID terms related to nodes 1.2, 1, 2, and 12 of the lattice decomposing I(X;123|3) can be nonzero. This is consistent with the fact that I(X;123|3)=I(X;12|3). The resulting decomposition of I(X;123|3) is in fact equivalent to the decomposition of I(X;12|3), which has only four terms. This is a desired property for the decomposition of I(X;123|3) because source 3 has no information after conditioning on target variable 3, and thus it has no information that can be classified as redundant, unique, or synergistic. Therefore, instead of assuming the nonnegativity of the terms Δ(X;α|3), we can alternatively directly impose that the PID of I(X;123|3) should equal the PID of I(X;12|3), as a desirable property of the PID terms. This already implies the fulfillment of the constraints of Equation (A1). This extra requirement is specific of the case $X^{\prime }=S^{\prime }$, since for the case I(X;123|X(3)), with X(3) a part of the target functionally determined by 3, we have H(3|X(3))>0. The logic of imposing the equivalence between the decomposition of I(X;123|3) and I(X;12|3) can be further understood examining the mapping of the four PID terms that can be nonzero (1.2, 1, 2, and 12) of the decomposition of I(X;123|3) (Figure A1B) to the PID terms of the decomposition of I(X;123) (Figure A1A). These four terms are mapped to different sums of terms that represent all the information other than I(X;3), which is not available after the conditioning on 3. Finally, the only synergistic term that can be nonzero in Figure A1B is 12, which is consistent with Equation (12), since the collection 12 is the only above the nodes corresponding to single primary sources such that none of its sources contains 3. There is no synergy between any source W⊆S−3 and the primary source 3 because W⊆S−3 already can provide, in combination with the conditioning copy of 3 from the target, any information obtained by jointly having {W,3}. Furthermore, also the terms 2.13 and 1.23 vanish in Figure A1B. This is consistent with the expression of $\Delta_{d}\left(X;i.jk\right)$ in Table 4, although Equation (12) does not concern these terms.

We can now resume the general case. In general, the equality of Equation (11) between the synergistic terms of I(X;S) and $I(X;S|X^{\prime })$ cannot be further specified. For the case of $X^{\prime }=S^{\prime }$, $I\left(X;S|X^{\prime }\right)=I\left(X;S|S^{\prime }\right)$. In this case, since $I\left(X;S|S^{\prime }\right)=I\left(X;S-S^{\prime }|S^{\prime }\right)$, we can impose as a desirable property of the PID of $I(X;S|S^{\prime })$ that it is equivalent to the PID of $I(X;S-S^{\prime }|S^{\prime })$. This is because $H(S^{\prime }|S^{\prime })=0$, and thus the sources in $S^{\prime }$ have no information after conditioning on their target copies. We can now see that this extra condition guarantees the consistency of the constraints of Equation (11) and Equation (12) proposed following the conceptual formulation of the strong axiom. Because the sources in S’ do not appear as sources in $I(X;S-S^{\prime }|S^{\prime })$, the PID of $I(X;S|S^{\prime })$ can only be equal to the one of $I(X;S-S^{\prime }|S^{\prime })$ if all PID terms $\Delta (X;S|S^{\prime })$ corresponding to a collection with a source *A* containing $S_{i}\in S^{\prime }$ vanish. This corresponds to the condition $\exists A\in \alpha ,S^{\prime }\cap A=\emptyset$ of Equation (12). Accordingly, since $\Delta \left(X-S^{\prime };S|S^{\prime }\right)=\Delta \left(X;S|S^{\prime }\right)$, Equation (12) is subsumed by Equation (11). The equivalence of the PID of $I(X;S-S^{\prime }|S^{\prime })$ and the PID of $I(X;S|S^{\prime })$ is also consistent with the constraints of the form of Equation (A1) related to Equation (9). This is because, as shown for the example above, given the partial ordering relations of Equation (8), canceling the PID terms of all collections containing a source A such that $S^{\prime }\cap A=\emptyset$ is equivalent to canceling the PID of all collections within $\left(↓\left\{W,S^{\prime }\right\}\right)-\left(↓W\right)$, for any $W\subseteq S-S^{\prime }$. Altogether, although in Section 3, we proposed the constraints of Equation (11) and of Equation (12) as separately following from the conceptual formulation of the strong axiom, if imposing as a further desirable condition to the PID of $I(X;S|S^{\prime })$ its equivalence to the PID of $I(X;S-S^{\prime }|S^{\prime })$, Equation (12) can be derived from Equation (11).

## Appendix B. Alternative Partitioning Orders for the Bivariate Decomposition with Target-Source Overlap

We here derive in more detail the alternative expressions for the unique and redundant information terms collected in Table 1, which are obtained applying the other mutual information partitioning order of Equation (15b). Using the relation decomposing conditional mutual information into unique information and synergy, we get:

(A2) $$\begin{array}{cc} I(X;1\backslash 2) & =I(X;1|2)-I(X;12\backslash 1,2)\\ & =I(X\cap 12;1|2)+I(X-12;1|2,X\cap 12)-I(X-12;12\backslash 1,2). \end{array}$$

This leads to express the unique information of 1 as:

(A3) $$I\left(X;1\backslash 2\right)=\left\{\begin{array}{c} I(X-12;1\backslash 2)\;\;\mathrm{if}\;X\cap 1=\emptyset \\ H(1|2)-I(X-12;12\backslash 1,2)\;\;\mathrm{if}\;X\cap 1\neq \emptyset \end{array}\right..$$

In this case, the unique information is separated into nonadditive terms and involves the synergy about X−12. This cross-over may seem at odds with the expression obtained with the other partitioning order (Equation (18)), but on the contrary it reflects the internal consistency of the relations between the information-theoretic quantities: Equations (18) and (A3) coincide if 1 is not part of the target. For X∩1≠∅, their equality:

(A4) H(1|2)−I(X−12;12\1,2)=I(X−12;1\2)+H(X∩1|2,X−12)

is consistent with the definition I(X−12;1|2)=H(1|2)−H(1|2,X−12), taking into account that conditional information is the sum of the unique and synergistic components.

Proceeding as with the other partitioning order, once we have the expression of the unique information we can use the relation with the mutual information to determine redundancy:

(A5) $$I\left(X;1.2\right)=\left\{\begin{array}{c} I(X-12;1.2)\;\;\mathrm{if}\;X\cap 12=\emptyset \\ I(1;2)+I(X-12;12\backslash 1,2)\;\;\mathrm{if}\;X\cap 12\neq \emptyset \end{array}\right..$$

Also here, internal consistency with Equation (19) holds. In particular, the equality:

(A6) I(1;2)+I(X−12;12\1,2)=I(X−12;1.2)+I(1;2|X−12)

reflects that:

(A7) $$\begin{array}{cc} C(X-12;1;2) & =I(1;2)-I(1;2|X-12)\\ & =I(X-12;1.2)-I(X-12;12\backslash 1,2) \end{array}$$

because the co-information is invariant to permutations (Equation (5)) and also corresponds to the difference of the redundancy and synergistic PID components.

Also following the strong axiom the alternative partitioning order, in this case the one considering first stochastic dependencies with the non-overlapping target variables, can be derived. With overlap, Equation (14) implies that I(X;1\2)=I(X;1|2). For the unique information, we get:

(A8) I(X;1\2)=I(X−12;1\2)+I(X−12;12\1,2)+H(X∩1|2,X−12),

and for the redundancy:

(A9) I(X;1.2)=I(X−12;1.2)+I(1;2|X−12)−I(X−12;12\1,2).

Like with the weak axiom, internal consistency holds for the expressions obtained with the two partitioning orders.

## Appendix C. The Fulfillment of the Strong Axiom by the Measures *SI*, and *I_red_*

We first show that the maximum conditional entropy-based decomposition of [36] conforms to the strong axiom:

**Proposition** **A1.** The PID associated with the redundancy measure SI [36] conforms to the synergy strong stochasticity axiom for any system with a target X containing a subset {X(1),X(2)} of variables that are completely determined by one of the primary sources 1 or 2.

**Proof.** Consider a target *X* and two sources 1 and 2. The measure of synergy associated to SI is defined as:

(A10) $$CI\left(X;1,2\right)=I\left(X;1,2\right)-\min_{Q\in \Delta (p)}I_{Q}\left(X;1,2\right),$$

where information is minimized within the family of distributions Δ(p) that preserves the marginals p(X,1) and p(X,2). Define X(i), as the subset of variables in *X* that are completely determined by the source i∈{1,2}. Define $X^{*}=X-\left\{X\left(1\right),X\left(2\right)\right\}$. We can now re-express CI(X;1,2) as:

(A11) $$\begin{array}{cc} CI(X;1,2) & =I\left(X^{*}X\left(1\right)X\left(2\right);1,2\right)-\min_{Q\in \Delta (p)}I_{Q}\left(X^{*}X\left(1\right)X\left(2\right);1,2\right),\\ & =\left[I\left(X\left(1\right)X\left(2\right);1,2\right)+I\left(X^{*};1,2|X\left(1\right),X\left(2\right)\right)\right]-\min_{Q\in \Delta (p)}\left[I_{Q}\left(X\left(1\right)X\left(2\right);1,2\right)+I_{Q}\left(X^{*};1,2|X\left(1\right),X\left(2\right)\right)\right]. \end{array}$$

We now show that $I_{Q}\left(X\left(1\right)X\left(2\right);1,2\right)=I\left(X\left(1\right)X\left(2\right);1,2\right)$∀Q∈Δ(p):

(A12) $$\begin{array}{cc} I_{Q}\left(X\left(1\right)X\left(2\right);1,2\right) & =I_{Q}\left(X\left(1\right);1,2\right)+I_{Q}\left(X\left(2\right);1,2|X\left(1\right)\right)\\ & =I_{Q}\left(X\left(1\right);1\right)+I_{Q}\left(X\left(1\right);2|1\right)+I_{Q}\left(X\left(2\right);2|X\left(1\right)\right)+I_{Q}\left(X\left(2\right);1|2,X\left(1\right)\right)\\ & =I_{Q}\left(X\left(1\right);1\right)+I_{Q}\left(X\left(2\right);2|X\left(1\right)\right)=I\left(X\left(1\right);1\right)+I\left(X\left(2\right);2|X\left(1\right)\right), \end{array}$$

where the last equality holds because p(X(1),1) and p(X(2),2,X(1)) are preserved in Δ(p). This means that Equation (A11) can be further simplified to:

(A13) $$\begin{array}{cc} CI(X;1,2) & =I\left(X^{*};1,2|X\left(1\right),X\left(2\right)\right)-\min_{Q\in \Delta (p)}I_{Q}\left(X^{*};1,2|X\left(1\right),X\left(2\right)\right)\\ & =CI(X^{*};1,2|X\left(1\right),X\left(2\right)). \end{array}$$

This corresponds to the synergy of the bivariate decomposition of $I(X^{*};1,2|X\left(1\right),X\left(2\right))$, in agreement with the strong axiom formulated in Equation (11). ☐

We now show that the measure $I_{red}$ also complies with the strong axiom, at least for a wider class of systems than the one concerned by the identity axiom:

**Proposition** **A2.** The PID associated with the redundancy measure $I_{red}$ [35] conforms to the synergy strong stochasticity axiom for any system with a target X containing both primary sources 1 and 2.

**Proof.** Consider a target *X* and two sources 1 and 2. The work in [35] defined the measure of redundancy based on information projections in the space of probability distributions. The projection of p(X|2) in the space of distributions of p(X|1), named $p_{2↘1}\left(X|2\right)$, is defined as the distribution

(A14) $$q\left(X\right)=\sum_{1}\alpha \left(1\right)p\left(X|1\right)$$

with α(1) being a probability distribution optimized such that $p_{2↘1}\left(X|2\right)=\arg\min_{q}\mathrm{KL}\left(p\left(X|2\right);q\left(X\right)\right)$. That is, $p_{2↘1}\left(X|2\right)$ minimizes the Kullback-Leibler divergence with p(X|2) in the space of the probability distributions defined by Equation (A14). The corresponding projected information of 2 onto 1 with respect to *X* is defined as:

(A15) $$I_{2↘1}\left(X;2\right)=\sum_{x,2}p\left(x,2\right)\log\frac{p_{2↘1}\left(x|2\right)}{p(x)}.$$

Redundancy is then defined as $I\left(X;1.2\right)=\min\{I_{2↘1}\left(X;2\right),I_{1↘2}\left(X;1\right)\}$. We now examine $I_{2↘1}\left(X;2\right)$ for the case in which 1 is part of *X*. In particular define X={Y,1}. For this case the distributions q(X) correspond to:

(A16) $$q\left(X\right)=\sum_{1^{\prime }}\alpha \left(1^{\prime }\right)p\left(Y,1|1^{\prime }\right)=\alpha \left(1\right)p\left(Y|1\right).$$

The KL-divergence is then:

(A17) $$\begin{array}{cc} \mathrm{KL}(p(Y,1|2);q(Y,1)) & =\sum_{y,1}p\left(y,1|2\right)\log\frac{p(y,1|2)}{\alpha (1)p(y|1)}\\ & =\sum_{1}p\left(1|2\right)\sum_{y}p\left(y|12\right)\log\frac{p(y|12)}{p(y|1)}+\sum_{1}p\left(1|2\right)\log\frac{p(1|2)}{\alpha (1)}. \end{array}$$

The first summand does not depend on α(1) and is thus constant in the minimization space. The second summand is zero if α(1)=p(1|2), which minimizes the divergence. Accordingly, with this value of α(1) into Equation (A16), $p_{2↘1}\left(X|2\right)=p\left(1|2\right)p\left(Y|1\right)$. Plugging this distribution into Equation (A15) for X={Y,1}, we have:

(A18) $$I_{2↘1}\left(Y,1;2\right)=\sum_{y,1,2}p\left(y,1,2\right)\log\frac{p(1|2)p(y|1)}{p(y,1)}=\sum_{1,2}p\left(1,2\right)\log\frac{p(1|2)}{p(1)}=I\left(1;2\right).$$

Now, for the case considered in this proposition, both 1 and 2 are part of *X*. We can thus repeat the derivation above to find that $I_{2↘1}\left(X;2\right)=I_{1↘2}\left(X;1\right)=I\left(1;2\right)$, and hence I(X;1.2)=I(1;2). This proves that for this case the PID is equal to the one obtained with the strong axiom. ☐

Note that the proof presented above for $I_{red}$ only contemplates the case in which both primary sources are part of the target, a more specific case than for the proofs presented for SI and $I_{dep}$ in Section 4.4, which show that those measures comply with the strong axiom whenever one of the primary sources is part of the target. This is because, when 1 is part of *X*, the information projection $I_{2↘1}\left(X;2\right)=I\left(1;2\right)$, but the information projection $I_{1↘2}\left(X;1\right)$ is in general not equal to I(1;2) unless 2 is also part of *X*. Despite of its more reduced scope, the proposition for $I_{red}$ encompasses relevant systems as the ones involved in the reduction of target 123 to 12 when 3 is a deterministic function of the primary sources 1 and 2.

## Appendix D. The Relation between the Constraints of Equations (11) and (12) for *SI*, *I_dep_*, and *I_red_*

In Section 4.3, we have shown that SI and $I_{dep}$ fulfill Equation (12) when X∩12≠∅, and in Appendix C that $I_{red}$ fulfills Equation (12) when X∩12=12. We now show that in these cases Equation (11) is consistently fulfilled. We start with SI:

**Proposition** **A3.** In the case of X∩12≠∅, the PID associated with the redundancy measure SI fulfills Equation (11) consistently with Equation (12).

**Proof.** It suffices that X∩12≠∅ so that the joint distribution p(X,1,2) is preserved within the family of distributions Δ(p). Given Equation (A13), this implies that the synergy CI(X;1,2) vanishes, in agreement with Equation (12). ☐

We now prove this consistency for $I_{dep}$:

**Proposition** **A4.** In the case of X∩12≠∅, the PID associated with the redundancy measure $I_{dep}$ fulfills Equation (11) consistently with Equation (12).

**Proof.** Consider $X^{\prime }=X\cap 12$ like in Equation (11) for the case that $X^{\prime }=S^{\prime }$. If X∩12≠∅, following the definition of unique information of [43] described in Section 4.3 (according to Appendix B of [43]), the unique information of 2 with respect to 1 in the PID decomposing $I(X;12|X^{\prime })$ can be expressed as:

(A19) $$I(X;2\backslash 1|X^{\prime })=\min\left\{I_{X2,1}\left(X;2|X^{\prime }\right),I_{X2,12}\left(X;2|X^{\prime }\right),I_{X1,X2}\left(X;2|X^{\prime }\right),I_{X1,X2,12}\left(X;2|X^{\prime }\right)\right\},$$

where $I_{X1,X2}\left(X;2|X^{\prime }\right)$ indicates the conditional mutual information for the maximum entropy distribution preserving p(X,1) and p(X,2), and analogously for the other mutual informations. If $1\in X^{\prime }$, p(X,1,2) is preserved for all the maximum entropy distributions. If $2\in X^{\prime }$, all the mutual informations vanish. Accordingly, it suffices X∩12≠∅ so that $I(X;2\backslash 1|X^{\prime })=I\left(X;2|X^{\prime }\right)$. Because, from Equation (4), $I\left(X;2|1,X^{\prime }\right)=I(X;2\backslash 1|X^{\prime })+I(X;12\backslash 1,2|X^{\prime })$, this implies that $I(X;12\backslash 1,2|X^{\prime })=0$ in agreement with Equation (12). ☐

We now prove this consistency for $I_{red}$:

**Proposition** **A5.** In the case of X∩12=12, the PID associated with the redundancy measure $I_{red}$ fulfills Equation (11) consistently with Equation (12).

**Proof.** Given Equation (6), the synergistic term of the PID of $I(X;12|X^{\prime })$ can be expressed as:

(A20) $$I(X;12\backslash 1,2|X^{\prime })=I\left(X;1|2,X^{\prime }\right)-I\left(X;1|X^{\prime }\right)+I\left(X;1.2|X^{\prime }\right).$$

For $X^{\prime }=X\cap 12=12$, both $I(X;1|2,X^{\prime })$ and $I(X;1|X^{\prime })$ vanish. From the definition of the projected information of 2 onto 1 with respect to *X* conditioned on $X^{\prime }$, analogous to Equation (A15), $I_{2↘1}\left(X;2|X^{\prime }\right)=0$ for $X^{\prime }=12$. Similarly, also $I_{1↘2}\left(X;1|X^{\prime }\right)$ vanishes. Accordingly, also $I(X;1.2|X^{\prime })=0$, and hence $I(X;12\backslash 1,2|X^{\prime })$ vanishes in agreement with Equation (12). ☐

## Appendix E. Derivations of the Trivariate Decomposition with Target-Source Overlap

We here derive in more detail the trivariate deterministic PID components. We start with the derivations following the weak stochasticity axiom. If we consider the unique information of one primary source with respect to the other two, for example I(X;3\12), we have that:

(A21) $$\begin{array}{cc} I(X;3\backslash 12) & =\Delta (X;3)\\ & =I\left(X;3|12\right)-\left[\Delta (X;123)+\Delta (X;13)+\Delta (X;23)+\Delta (X;13.23)\right]. \end{array}$$

The weak axiom imposes for the trivariate case that synergy deterministic components upper than the single source nodes have to be zero (Equation (27)). Accordingly, any deterministic component of I(X;3|12) has to be contained in Δ(X;3). Decomposing this conditional mutual information with the partitioning order that considers first the dependencies with the non-overlapping target variables:

(A22) I(X;3\12)=I(X−123;3\12)+H(X∩3|12,X−123),

and thus in general:

(A23) $$\Delta_{d}\left(X;i\right)=H\left(X\cap i|jk,X-ijk\right).$$

We now consider the conditional information of two primary sources given the third, for example:

(A24) $$\begin{array}{cc} I(X;23|1) & =I(X-123;23|1)+H(X\cap 23|1,X-123). \end{array}$$

The deterministic part H(X∩23|1,X−123) again can only be contained in the PID terms contributing to I(X;23|1) that are lower than the single source nodes. This means that it has to be contained in the terms:

(A25) $$\begin{array}{c} \Delta_{d}\left(X;2\right)+\Delta_{d}\left(X;3\right)+\Delta_{d}\left(X;2.3\right)+\Delta_{d}\left(X;3.12\right)+\Delta_{d}\left(X;2.13\right). \end{array}$$

Furthermore, this conditional entropy can be decomposed considering explicitly the part of the uncertainty associated with conditional entropies of the form of Equation (A23):

(A26) $$\begin{array}{c} H(X\cap 23|1,X-123)=H(X\cap 3|1,X-123)+H(X\cap 2|1,X\cap 3,X-123)\\ =H(X\cap 3|12,X-123)+I(2;X\cap 3)|1,X-123)+H(X\cap 2|1,X\cap 3,X-123). \end{array}$$

Accordingly, using the definition of the terms $\Delta_{d}\left(X;i\right)$ in Equation (A23) and combining Equations (A24) and (A25), we get the following equalities. First,

(A27) $$\Delta_{d}\left(X;i\right)+\Delta_{d}\left(X;i.j\right)+\Delta_{d}\left(X;i.jk\right)+\Delta_{d}\left(X;j.ik\right)=H\left(i|k,X-ijk\right)\;\mathrm{if}\;X\cap i\neq \emptyset ,$$

and second:

(A28) $$\Delta_{d}\left(X;i.j\right)+\Delta_{d}\left(X;i.jk\right)+\Delta_{d}\left(X;j.ik\right)=I\left(i;j|k,X-ijk\right)\;\;\mathrm{if}\;X\cap i\neq \emptyset .$$

Like in the expressions of the deterministic PID components in the tables of Section 4.1 and Section 5.1, we here for simplicity indicate the equalities that hold when the primary source *i* overlaps with the target. The symmetries of each $\Delta_{d}\left(X;\beta \right)$ term indicate when it can be nonzero. For example, $\Delta_{d}\left(X;i.j\right)$ is constrained by an equality of the form of Equation (A28) both if *i* or *j* overlap with the target.

Finally, we consider also how an unconditional mutual information is decomposed in PID terms. For example, again using the partitioning order that considers first stochastic target-source dependencies, we have:

(A29) $$\begin{array}{cc} I(X;3) & =I(X-123;3)+I(X\cap 123;3|X-123)\\ & =I(X-123;3)+H(X\cap 3|X-123)\;\;\mathrm{if}\;X\cap 3\neq \emptyset . \end{array}$$

When 3 is part of the target the deterministic part of this information has to be contained in the nodes reached descending from 3, and thus in general:

(A30) $$\sum_{\beta \in ↓i}\Delta_{d}\left(X;\beta \right)=H\left(i|X-ijk\right)\;\;\mathrm{if}\;X\cap i\neq \emptyset .$$

Combining Equation (A30) with Equation (A27), we get that:

(A31) $$\begin{array}{cc} \Delta_{d}\left(X;i.j\right)+\Delta_{d}\left(X;i.j.k\right)-\Delta_{d}\left(X;k.ij\right) & =I(i;j|X-123)\;\;\mathrm{if}\;X\cap i\neq \emptyset \\ & =H(i|X-123)-H(i|j,X-123). \end{array}$$

Altogether, from Equations (A23), (A27), (A28), (A30), and (A31), we can proceed to obtain expressions of the PID terms as a function of mutual informations and entropies. Doing so, the rest of PID terms remain as a function also of the terms $\Delta_{d}\left(X;i.jk\right)$. These terms can be understood by comparing the trivariate decomposition and a bivariate decomposition with only sources *j* and *k*. For the latter, if *i* is part of the target, I(i;jk\j,k) quantifies a stochastic synergistic contribution, because *i* is not a source. Conversely, in the trivariate decomposition *i* is a source and this information is now redundant with the information provided by variable *i* itself. This means that we can identify $\Delta_{d}\left(X;i.jk\right)$ by comparing synergy between these two decompositions. For example, for the bivariate decomposition of I(X;12), 3 is not a source and according to the weak axiom synergy can provide information about the non-overlapping part of the target, which can comprise 3. Moving to the trivariate case by adding 3 as a source this synergy stochastic component becomes redundant to information source 3 has about itself, and thus:

(A32) $$\begin{array}{cc} I(X;12\backslash 1,2) & =I(X-12;12\backslash 1,2)\\ & =I\left(X-123;12\backslash 1,2\right)+\left[I(X-12;12\backslash 1,2)-I(X-123;12\backslash 1,2)\right]. \end{array}$$

In general, this means that these types of PID terms can be quantified as:

(A33) $$\begin{array}{c} \Delta_{d}\left(X;i.jk\right)=I\left(X-jk;jk\backslash j,k\right)-I\left(X-ijk;jk\backslash j,k\right). \end{array}$$

These terms are non-negatively defined, because according to the axiom adding a new source can only reduce synergy. After calculating these terms we can obtain all the expressions collected in Table 3.

For the strong stochasticity axiom, instead of repeating all the derivations we proceed by arguing about what has to change with respect to the decomposition obtained for the weak axiom. Changes originate from the difference in the constraints that both versions of the axiom impose on the existence of synergistic components and from the alternative mutual information partitioning order that leads to an additive separation of stochastic and deterministic PID components depending on the axiom. With the strong axiom this additive separation is reached using the partitioning order that first considers deterministic target-source dependencies. This means that the conditioning of entropies and mutual informations on X−ijk will in this case not be present. Moreover, since the strong axiom restricts also synergy with the non-overlapping target variables, even a stochastic component of I(X;12\1,2) can only be nonzero if 3, but neither 1 or 2, overlap with the target. Since once further adding 3 to the sources any synergistic component should be zero, the expression of the terms $\Delta_{d}\left(X;i.jk\right)$ in Equation (A33) is reduced to I(i;jk\j,k) when only *i* overlaps with *X*, and to zero otherwise. Implementing these two modifications, the expressions of Table 4 are obtained from the ones of Table 3.

## Appendix F. The Counterexample of Nonnegativity of Bertschinger et al. (2012), Rauh et al. (2014), and Rauh (2017)

We here describe in more detail the previous proofs of existence of negative terms for the trivariate PID of I(123;1,2,3), with 3 being the output of the XOR systems and 1 and 2 the inputs. These proofs show that, if preserving the original axioms of [34], it suffices to add the identity axiom, or even just the independent identity property, to originate negative terms. In these previous studies the terms I(123;i.j) were calculated assuming that redundancy is invariant to the reduction of 123 to 12, that is, that I(123;i.j)=I(ij;i.j). This invariance was not motivated in terms of what is expected from the notion of redundancy, but was accepted as inherited from the fact that it holds for the mutual information. We have now indicated that, given its implications regarding the assignment of information identity, it should be considered as an assumption at the same level of the ones more explicitly discussed in the proofs.

Given the equality I(123;i.j)=I(ij;i.j), by the independent identity property I(ij;i.j)=I(i;j)=0 because all three variables of the XOR system are independent. Furthermore, the terms I(123;i.jk) were determined by the monotonicity axiom [41] as I(123;i.ijk)=I(123;i)=1, or by the identity axiom [44] as I(i,jk;i.jk)=I(i;jk)=1. This means that the minimal extra assumptions, apart from the original axioms, are the independent identity property, as pointed out by [41], and the invariance under the reduction of 123 to 12.

Accordingly, from the nodes reachable descending from I(123;12.13.23), all redundancies are determined except I(X;1.2.3) and I(123;12.13.23) itself. Because the objective of the proof was only to show that a negative term has to exist, and not to actually calculate each PID term, [41,42,44] considered that I(X;1.2.3) vanishes, which preserves the monotonicity of I(X;1.2.3)≤I(X;i.j)=0. Given that, they determined a bound I(123;12.13.23)≤−1. In fact, one may argue that I(X;1.2.3) can be negative, which still preserves the monotonicity of I(X;1.2.3)≤I(X;i.j)=0, and already would lead to the existence of a negative term. In particular, I(X;1.2.3) can be negative if it is compensated by the terms Δ(X;i.j) being positive and of equal magnitude, so that I(X;i.j)=0. This is actually what we find in Figure 4B with the strong axiom, and this decomposition is completely compatible with the values I(X;i,j)=0 and I(X;i.jk)=1 determined in these proofs using the axioms as described above. Taking this into account, we see that these proofs work as proofs of existence of at least one negative term, but this negative term can either be Δ(X;1.2.3) or Δ(123;12.13.23).
